# METTL3 mediates m6A methylation of LCN2 through IGF2BP3 to promote ferroptosis in chronic obstructive pulmonary disease

**DOI:** 10.1186/s41065-025-00628-9

**Published:** 2025-12-26

**Authors:** Fang Chen, Dan Liu, Zuoquan Zhu, Da Chen

**Affiliations:** 1 Department of Geriatrics, Jingzhou First People’s Hospital, No.55, Jianghan North Road, Shashi District, Jingzhou, 434000 China; 2 Department of General Internal Medicine, Jingzhou First People’s Hospital, No.55, Jianghan North Road, Shashi District, Jingzhou, 434000 China

**Keywords:** COPD, LCN2, METTL3, M6A, Ferroptosis, IGF2BP3

## Abstract

**Background:**

Chronic obstructive pulmonary disease (COPD) is a chronic inflammatory lung disease characterized by persistent airflow obstruction. Studies have shown that Lipocalin 2 (LCN2) and Methyltransferase-like protein 3 (METTL3) are involved in COPD progression. However, whether LCN2 and METTL3 jointly regulate the progression of COPD and the molecular mechanism are still unclear.

**Methods:**

Human lung microvascular endothelial cells (HMVECs) were treated with Cigarette smoke extract (CSE) to construct an in vitro cell model of COPD. Then, cell viability and apoptosis were detected by CCK-8 and flow cytometry. Meanwhile, the levels of IL-6, TNF-α, ROS, Fe^2+^, MDA, and GSH were measured by ELISA and the corresponding kits. Bioinformatics analysis was used to predict the m6A methylation modification sites on LCN2 mRNA. Besides, the methylation modification level of LCN2 was monitored by MeRIP. The binding of METTL3 and IGF2BP3 to LCN2 was verified by RIP. And the stability of LCN2 mRNA was analyzed by actinomycin D. The role of LCN2 in COPD in vivo model was verified by constructing an in vivo mouse model.

**Results:**

Silencing LCN2 could effectively reduce CSE induced HMVECs injury and ferroptosis. METTL3 stabilized LCN2 expression through m6A methylation. IGF2BP3 could bind to LCN2 and stabilize its expression. METTL3 inhibitor STM2457 could restore CSE induced HMVEC injury and ferroptosis. Additionally, overexpression of LCN2 could also reverse the effect of METTL3 silencing on CSE-induced HMVEC injury. METTL3 inhibited the NRF2/SLC7A11/GPX4 pathway by stabilizing LCN2. Moreover, knockdown of LCN2 alleviated lung injury and inflammatory response in COPD mice and activate NRF2/SLC7A11/GPX4 signaling pathway.

**Conclusion:**

This study confirmed that METTL3/IGF2BP3 enhanced the stability of LCN2 and hindered the NRF2/SLC7A11/GPX4 pathway through m6A methylation modification, thereby aggravating the progression of COPD. It provides a new direction for the study of the mechanism of COPD, identifies key molecules such as METTL3 and IGF2BP3, and lays a foundation for the development of COPD treatment strategies targeting m6A modification or NRF2 pathway.

**Supplementary Information:**

The online version contains supplementary material available at 10.1186/s41065-025-00628-9.

## Introduction

Chronic obstructive pulmonary disease (COPD) stands as a pressing global public health concern, marked by its high rates of morbidity and mortality [1]. This condition is primarily defined by incomplete and reversible airflow limitation, a hallmark intimately linked to the lungs’ abnormal inflammatory response to exposure to harmful gases or particles [2, 3]. The progression of COPD encompasses intricate molecular mechanisms. Patients with COPD often show dysfunction of human lung microvascular endothelial cells (HMVECs) [4]. Vascular remodeling is one of the core pathological features of COPD, and it is also the key cause of “pulmonary hypertension” and “right heart failure” (late complications of COPD) in patients with COPD. The essence of vascular remodeling is the dysfunction of pulmonary microvascular endothelial cells [5]. Over the years, ferroptosis, as an emerging and unique iron-dependent regulated way of cell death, has attracted extensive scientific attention in the pathological process of COPD [6, 7]. The mechanism of ferroptosis mainly includes the increase of iron accumulation, the production of free radicals, and the accumulation of lipid peroxides [8]. Persistent inflammatory stimulation and oxidative stress state provide favorable conditions for the occurrence of ferroptosis in COPD’s pathological environment [9, 10]. However, the specific regulatory mechanisms of ferroptosis in COPD are not fully understood.

N6-methyladenosine (m6A) represents the most ubiquitous RNA modification observed in eukaryotic mRNAs [11]. The dynamic changes of m6A modification are intricately linked to a multitude of biological processes, encompassing cell differentiation, development, brain function, as well as disease occurrence [12]. In the last few years, numerous studies demonstrated that abnormalities of m6A modification are directly related to a wide array of diseases, ranging from cancer to neurodegenerative disorders and cardiovascular ailments [13–15]. It is worth noting that the emergence and progression of COPD are also markedly influenced by m6A modification [16]. One study disclosed that PM2.5 triggered pulmonary microvascular damage in individuals with COPD through methyltransferase-like protein 16 (METTL16)-mediated m6A methylation modification [17]. Xie et al. demonstrated that zinc finger CCCH-type containing 13 (ZC3H13) utilized m6A modification to enhance the expression level of integrin subunit alpha 6 (ITGA6) and the stability of its mRNA, which affected human bronchial epithelial cells (HBECs) inflammation and fibrosis and further aggravated the progression of COPD [18]. Methyltransferase-like protein 3 (METTL3) serves as the pivotal subunit within the m6A methyltransferase complex, having an influence on the formation of m6A modifications. In various biological processes, METTL3 mediates m6A methylation modifications of specific genes, thereby influencing the expression of target genes and cellular functions [19, 20].

Lipocalin 2 (LCN2) is a multifunctional siderophore protein that participates in the inflammatory response, which is notably upregulated in COPD patient’s lung tissues [21]. LCN2 regulates intracellular iron homeostasis by binding siderophore, affecting iron-dependent enzyme activities and REDOX reactions [22]. In addition, LCN2 induced ferroptosis by promoting Fe^2+^ accumulation and inhibiting the SLC7A11/GPX4 antioxidant axis via activating JNK pathway [23]. Recent research confirmed that LCN2 not only acted in regulating inflammatory responses but might also contributed to ferroptosis by influencing various cellular processes such as iron metabolism and lipid peroxidation [24, 25]. Most importantly, Increased LCN2 has been reported to be a molecular feature of COPD-associated lung adenocarcinoma [26]. However, the specific regulatory mechanism of LCN2 on ferroptosis in COPD, especially whether it is regulated by m6A methylation modification, is rarely elucidated.

Cigarette smoke exposure and air pollution are universally acknowledged as the most prevalent environmental risk factors for the development of COPD [27]. Studies have shown that Cigarette smoke extract (CSE) has the capacity to stimulate macrophages, inducing them to secrete inflammatory mediators, thereby promoting the progression of COPD [28, 29]. Therefore, in this study, HMVECs were treated with CSE to establish an in vitro cell model mimicking the vascular endothelial dysfunction associated with COPD. At the same time, in combination with the in vivo COPD model, this study aimed to delve into and elucidate whether METTL3 regulated the ferroptosis of HMVECs induced by CSE through mediating the m6A methylation modification of LCN2, thereby aggravating the progression of COPD.

## Materials and methods

### Cell culture

Human pulmonary microvascular endothelial cells HMVEC (Item No. SNP-H053, SUNNCELL, Wuhan, China) were maintained in ECM (Item No. YZ-1001, Solarbio, Beijing, China) containing 1% endothelial growth factor (Item No. 91516ES60, Yeasen, Shanghai, China), 5% fetal bovine serum (FBS) (Gibco, Grand Island, NY, USA), 5000 U/mL penicillin- streptomycin (Item No. 15070063, Invitrogen, Carlsbad, CA, USA), nd were maintained with 5% CO_2_ at 37℃.

### Cell transfection

Targeted LCN2 (si-LCN2), METTL3 (si-METTL3), IGF2BP1 (si-IGF2BP1), IGF2BP2 (si-IGF2BP2), IGF2BP3 (si-IGF2BP3), METTL14 (si-METTL14), METTL16 (si-METTL16), FTO (si-FTO), NSUN2 (si-NSUN2), YTHDF1 (si-YTHDF1), YTHDF2 (si-YTHDF2), YTHDF3 (si-YTHDF3), and YTHDC1 (si-YTHDC1) siRNAs were all designed and synthesized by GenePharma (Shanghai, China), and the non-targeting siRNA (si-NC) was served as the negative control. LCN2 gene or METTL3 gene were cloned into pcDNA3.1 plasmid (Invitrogen) to construct LCN2 overexpression vector (OE-LCN2) and METTL3 overexpression vector (OE-METTL3), and the empty pcDNA3.1 plasmid was used as the negative control. The siRNAs and overexpression vector were introduced into HMVECs utilizing Lipofectamine 2000 (Item No. 11668500, Invitrogen), adhering strictly to the manufacturer’s guidelines.

### Preparation of CSE

After burning unfiltered standard cigarettes (containing 14 mg of carbon monoxide, 1.0 mg of nicotine, and 13 mg of tar per cigarette), which were thoroughly dissolved in 4 mL of PBS, resulting in the preparation of the original CSE (100% CSE) [30]. Subsequently, the CSE solution was filtered through a 0.22 μm filter (Invitrogen) and used within 30 min. The stock solution of CSE was then diluted with PBS to 1% CSE, 2% CSE, and 3% CSE to be used. HMVECs were exposed to the prepared CSE for 48 h, followed by subsequent experiments.

### Western blot (WB)

HMVECs were lysed and protein concentration was determined using the BCA Protein Assay Kit (Item No. P0010, Beyotime, Shanghai, China). Protein samples were subjected to SDS-PAGE to separate proteins in cell lysates. At the end of electrophoresis, they were transferred to PVDF membranes (Millipore, Billerica, MA, USA) and blocked with 5% skim milk (Item No. P0216-300 g, Beyotime) for 1 h at room temperature on a shaker. The PVDF membranes were washed and subsequently incubated with the corresponding primary antibodies overnight at 4℃. Then, they were incubated with the secondary antibodies for 2 h at room temperature. Finally, the BeyoECL Plus Kit (Item No. P0018S, Beyotime) was applied to visualize the measured proteins, and the Image J software was used to perform gray scale analysis of the protein bands. The primary antibodies used included Anti-lipocalin-2 antibody (1:1000, Item No. ab318209, Abcam, Cambridge, UK), Anti-METTL3 antibody (1:2000, Item No. ab240595, Abcam), Anti-IGF2BP1 antibody (1:1000, Item No. ab184305, Abcam), Anti-IGF2BP2 antibody (1:2000, Item No. ab124930, Abcam), Anti-IGF2BP3 antibody (1:1000, Item No. ab177477, Abcam), Anti-NRF2 antibody (1:1000, Item No. ab137550, Abcam), Anti-SLC7A11 antibody (1:1000, Item No. ab300667, Abcam), Anti-GPX4 antibody (1:1000, Item No. ab41787, Abcam), Anti-GAPDH antibody (1:1000, Item No. ab181602, Abcam). The secondary antibody was Goat Anti-Rabbit IgG H&L (HRP) (1:5000, Item No. ab6721, Abcam).

### Cell counting kit 8 (CCK-8)

HMVECs were inoculated in 96-well plates, and the corresponding CSE exposure and transfection treatments were performed according to the experimental requirements after the cells adhered to the wall. Then, cell viability was examined using the CCK-8 Kit (Item No. 40203ES60, Yeasen). 10 µL of CCK-8 reagent was added to each well, followed by gentle shaking to thoroughly mix. After that, the 96-well plates were carefully placed back into the incubator to react for 3 h. Finally, the absorbance values of each well were measured at a wavelength of 450 nm via a microplate reader.

### Flow cytometry

Cell apoptosis was monitored via flow cytometry using the Annexin V-FITC/PI Apoptosis Detection Kit (Item No. 40302ES50, Yeasen). After collecting the processed cells, they were washed with pre-cooled PBS and re-suspended in 100 µL of 1×Binding Buffer solution. 5 µL of Annexin V-FITC and 10 µL of Propidium Iodide (PI) solution were added to the suspension, and staining was performed at room temperature for 15 min. After staining, 400 µL of 1×Binding Buffer was added for dilution and mixing, and then the mixture was placed on ice. Finally, the processed cell samples were analyzed on the flow cytometer.

### Enzyme-linked immunosorbent assay (ELISA)

The levels of IL-6 and TNF-α were assessed using the Human IL-6 ELISA Kit (Item No. 97068ES96, Yeasen) and Human TNF-α ELISA Kit (Item No. 97072ES96, Yeasen) in strict accordance with the operating methods provided in the manufacturer’s instructions.

### Measurement of ferroptosis

The main characteristics of ferroptosis are the accumulation of intracellular iron ions and the production of Reactive oxygen species (ROS) leading to lipid peroxidation [31]. Therefore, the level of ROS, Fe^2+^ and malondialdehyde (MDA) in HMVECs can indirectly reflect the progression of ferroptosis. Detection of ROS levels was achieved by the Reactive Oxygen Species Assay Kit (Item No. 50101ES01, Yeasen). Specifically, HMVECs were co-incubated with DCFH-DA probe in the dark at 37℃ for 30 min. After that, the fluorescence intensity of ROS was directly observed and analyzed using the laser scanning confocal microscope. Immediately thereafter, the cells were digested, collected, and subjected to flow cytometry analysis to count the fluorescent cells. For the detection of Fe^2+^ and MDA levels, the Iron Assay Kit (Item No. ab83366, Abcam) and Lipid Peroxidation MDA Assay Kit (Item No. S0131S, Beyotime) were utilized and determined according to the operating instructions. GSH levels were monitored by the GSH and GSSG Detection Kit (Item No. S0053, Beyotime).

### Bioinformatics analysis

The RMbase (https://bioinformaticsscience.cn/rmbase/) and RMvar (https://rmvar.renlab.org/index.html) databases were employed to predict the presence of m6A methylation modification on the mRNA sequence of LCN2. In addition, the m6A methylation modification sites on the LCN2 mRNA were predicted by the SRAMP website (http://www.cuilab.cn/m6asiteapp/old).

### Real-time quantitative PCR (RT-qPCR)

HMVECs were treated with the TRIzol reagent (Invitrogen) to extract total RNA and reverse transcribed into cDNA using the Hifair^®^ AdvanceFast 1 st Strand cDNA Synthesis Kit (Item No. 11149ES60, Yeasen). RT-qPCR reactions were performed using specific primers (RiBoBio, Guangzhou, China) for the gene in the presence of Hieff UNICON^®^ ColorGPS qPCR SYBR Green Master Mix (Item No. 11188ES08, Yeasen) using cDNA as a template. Gene expression was quantified using the 2^−∆∆CT^ method with GAPDH as a control. The specific primer sequences used were as follows: LCN2: F: 5’-ATCTCGGGTGCCTCCCATTT-3’, R: 5’- CCAGCTCCCTCAATGGTGTT-3’. GAPDH: F: 5’-.

TTTTGCGTCGCCAGCC-3’, R: 5’- ATGGAATTTGCCATGGGTGGA-3’.

### Methylated RNA Immunoprecipitation (MeRIP)

The m6A methylation modification level of LCN2 was evaluated by MeRIP assay. Total RNA of HMVECs was extracted and purified using the Dynabeads™ mRNA Purification Kit (Item No. 61006, Invitrogen). The pre-washed Pierce™ Protein A/G Magnetic Beads (Item No. 88802, Thermo Fisher Scientific) were subsequently incubated with Anti-m6A antibody (Item No. ab208577, Abcam) or Rabbit IgG antibody (Item No. ab172730, Abcam) for 2 h at 4℃. The purified RNA was then mixed with antibody-conjugated magnetic beads and added to 1×immunoprecipitation buffer (Beyotime) containing RNase inhibitor (Item No. R0102-2kU, Beyotime). Proteinase K (Item No. ST535-100 mg, Beyotime) was added and the methylated mRNA was precipitated using glycogen, sodium acetate, and ethanol overnight at −80℃. Finally, the level of m6A methylation modification was analyzed by calculating the degree of enrichment by RT-qPCR reaction.

### RNA Immunoprecipitation (RIP)

To verify the enrichment effect of METTL3 on LCN2 mRNA, RIP assay was performed. In brief, HMVECs were washed three times with pre-cooled PBS solution to remove residual medium. Then, they were lysed with RIPA lysis buffer (Item No. P0013B, Beyotime) for 10 min under ice bath conditions, and the supernatant was collected by centrifugation. The supernatant was incubated with the Anti- METTL3 Antibody (Item No. ab195352, Abcam) and Anti-IgG antibody (Item No. ab172730, Abcam) for 2 h at 4℃. After that, pre-washed Protein A/G magnetic beads (Item No. 88802, Thermo Fisher Scientific) were added and the incubation was continued for 2 h at 4℃. After the elution step, the RNA was purified using the Dynabeads™ mRNA Purification Kit (Item No. 61006, Invitrogen), and finally the purified RNA was further analyzed and identified by RT-qPCR assay.

### RNA stability assay

HMVECs were seeded and transiently transfected accordingly, and were then treated with Actinomycin D (0.2 mmol/L) (Item No. ELS-BML-GR300-0005, Beyotime). The total RNA was collected at 0 h, 3 h, and 6 h after treatment for RT-qPCR to measure the levels of LCN2 mRNA.

### METTL3 inhibitor STM2457

STM2457 is a potent and selective inhibitor of RNA methyltransferase METTL3 with significant antitumor activity [32]. In this study, HMVECs were treated with STM2457 (20 µM) (MedChemExpress, New Jersey, USA) for 48 h to impair the m6A methylation ability of METTL3.

### Construction of an in vivo COPD model

The experimental animals used in this study were 6–8 weeks old female C57BL/6 mice purchased from Beijing SPF Biotechnology Co., Ltd. (Beijing, China). The mice were randomly divided into 4 groups (5 mice in each group): the Control group, the CS group, the CS + Ad-sh-NC group, and the CS + Ad-sh-LCN2 group. Mice in the CS + Ad-sh-NC group and the CS + Ad-sh-LCN2 group were subcutaneously injected with sh-NC or sh-LCN2 transfected HMVECs (5 × 10^6^ cells in 100 µL PBS) in advance. All the mice were pre-treated with lung lavage. Subsequently, the mice in each group were exposed to 4 CS for 1 h per day for 12 weeks using a special smoking chamber. All animal procedures were approved by the Jingzhou First People’s Hospital Animal Care and Use Committee.

### Validation of the COPD model

After the COPD model was established, the mice were fasted and water deprived for 6 h and then anesthetized with 1% sodium pentobarbital (Item No. 4390-16-3, Sigma-Aldrich, St. Louis, MO, USA). Then, the cervical trachea of the mice was isolated, and 0.9 mL of precooled sterile saline was extracted with a 1 mL syringe, and saline was slowly injected into the trachea (0.1 mL/s). After 5 s, the bronchoalveolar lavage fluid (BALF) was collected into a centrifuge tube, which could be repeatedly irrigated for 3 times. Then, the collected BALF was centrifuged, and the supernatants were separated and tested for IL-6 and TNF-α secretion by the corresponding kits (Yeasen). Cells at the bottom of the tube were resuspended in 500 µL of normal saline, and cells were counted using a hemacytometer plate.

### HE staining

The mice were sacrificed by cervical dislocation, and the intact lungs were removed by rapid thoracotomy. The lung tissues were rinsed with 4℃ pre-cooled normal saline and were fixed with 4% paraformaldehyde (Item No. 30525-89-4, Sigma-Aldrich) for 24 h. After that, they were dehydrated, cleared, embedded in paraffin, and sectioned into 4 μm thickness. The dried sections were deparaffinized using xylene (Item No. 1330-20-7, Sigma-Aldrich) and gradient concentrations of ethanol (Item No. 64-17-5, Sigma-Aldrich). The sections were placed in Harris hematoxylin solution (Item No. 517-28-2, Sigma-Aldrich) and stained for 10 min at room temperature. The sections were differentiated with 1% ethanol hydrochloride (Item No. C0163M, Beyotime) and immediately placed in 0.5% ammonia water (Item No. 1336-21-6, Sigma-Aldrich) to return blue for 30 s. They were subsequently stained in 0.5% eosin staining solution (Item No. C0109, Beyotime) at room temperature for 5 min. Finally, the sections were dehydrated and transparent, sealed with neutral gum (Item No. C0173, Beyotime) and observed under the light microscope.

### MASSON staining

For MASSON staining, the sections were deparaffinized to water following the same procedure as for HE staining. The sections were then stained in Weigert iron hematoxylin solution (Item No. HT1079, Sigma-Aldrich) for 15 min at room temperature before differentiation and reversion to blue and were then stained with 0.5% acid fuchsin staining solution (Item No. 3244-88-0, Sigma-Aldrich) for another 10 min. Subsequently, the sections were immersed in 1% phosphomolybdic acid solution (Item No. 12026-57-2, Sigma-Aldrich) for 10 min and directly transferred to 0.2% aniline blue staining solution (Item No. 28631-66-5, Sigma-Aldrich) for another 8 min. Rapid differentiation was performed using 0.5% glacial acetic acid solution (Item No. S0302, BIOSS, Beijing, China) for 2 min. Finally, the sections were dehydrated, transparent, and sealed. Alveolar tissue was observed under a light microscope.

### Statistical analysis

The data were analyzed in the GraphPad Prism 8.0.1 software and presented as mean ± standard deviation. The differences between the two groups were compared using Student’s *t*-test, and the Bonferroni method was employed to correct the difference. The Shapiro-Wilk normality test was used to test the normality. The comparison between multiple groups was analyzed via one-way or two-way analysis of variance, accompanied by a post hoc test with Turkey’s method. *P* < 0.05 was considered statistically significant.

## Results

### Silencing LCN2 reversed CSE induced HMVEC injury and ferroptosis

**Fig. 1:**
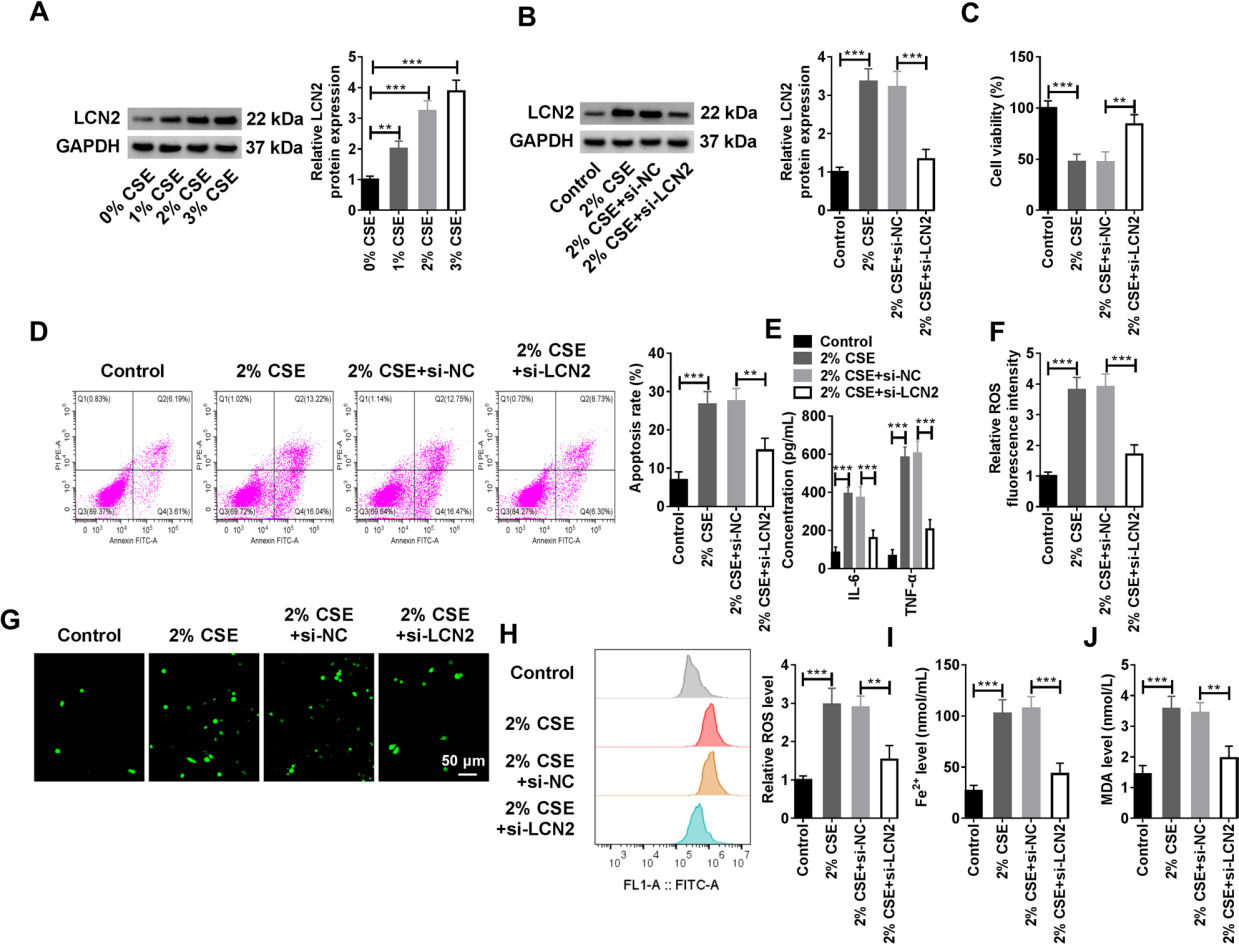
Effect of silencing LCN2 on CSE induced HMVEC injury and ferroptosis. **A** HMVECs were exposed to different concentrations of CSE (0%, 1%, 2%, and 3%), and the protein levels of LCN2 were detected by WB (Biological replicates, *N* = 3). (B-K) HMVECs were divided into the control group, 2% CSE group, 2% CSE + si-NC group, and 2% CSE + si-LCN2 group. **B** WB analysis of LCN2 protein levels in HMVECs (Biological replicates, *N* = 3). **C **The CCK-8 kit was used to monitor the viability of HMVECs (Biological replicates, *N* = 3). **D** Flow cytometry was used to analyze the apoptosis rate of HMVECs (Biological replicates, *N* = 3). **E** The levels of IL-6 and TNF-α were measured by the corresponding ELISA kits (Biological replicates, *N* = 3). **F **and **G** Immunofluorescence assay was used to analyze the level of ROS in HMVECs. **H** Flow cytometry was applied to analyze the level of ROS in HMVECs (Biological replicates, *N* = 3). **I **and **J** The levels of Fe^2+^ and MDA in HMVECs were determined by the corresponding kits (Biological replicates, *N* = 3). Statistical analysis of the data was performed using one-way ANOVA with Turkey’s post hoc test. **P < 0.01, ***P < 0.001

First, HMVECs were induced by 0%, 1%, 2%, and 3% CSE, and it was found that the protein level of LCN2 was increased in a CSE concentration-dependent manner (Fig. 1A). Considering that the protein level of LCN2 in HMVECs exposed to 2% CSE was significantly higher than that in the control group (0% CSE), 2% CSE was selected as the subsequent induction concentration of HMVECs. Subsequently, LCN2 was treated for loss of function, and the knockdown efficiency of LCN2 in HMVECs was examined by WB. Figure 1B revealed that the expression of LCN2 was markedly elevated in HMVECs exposed to CSE, whereas the protein level of LCN2 was notably reduced in HMVECs transfected with si-LCN2 compared to the si-NC-transfected group. The CCK-8 assay results indicated that the viability of HMVEC was distinctly repressed following exposure to CSE. However, when the expression of LCN2 was curbed, the cell viability was obviously restored (Fig. 1C). At this point, the apoptosis rate of the cells was monitored, suggesting that treatment with CSE specially elevated the apoptosis rate of HMVECs. However, upon LCN2 knockdown, the apoptosis rate of the cells was effectively suppressed (Fig. 1D). The ELISA measurements of inflammatory cytokines implicated that the introduction of CSE stimulated the release of IL-6 and TNF-α. Conversely, knockdown of LCN2 effectively blocked the levels of both IL-6 and TNF-α (Fig. 1E). Fluorescence intensity and flow cytometry analyses of ROS content in HMVECs proved that treatment with CSE led to an upregulation of ROS levels. In contrast, silencing LCN2 resulted in a downward trend in ROS levels (Fig. 1F-H). Besides, the increased levels of Fe^2+^ and MDA in HMVECs in response to CSE were also decreased when LCN2 was silenced (Fig. 1I and J). Collectively, CSE could upregulated LCN2 levels in HMVECs to hamper the activity and proliferation of HMVECs, promote cell apoptosis and the release of inflammatory factors IL-6 and TNF-α, as well as induce ferroptosis.

### METTL3 could mediate the m6A methylation of LCN2

**Fig. 2:**
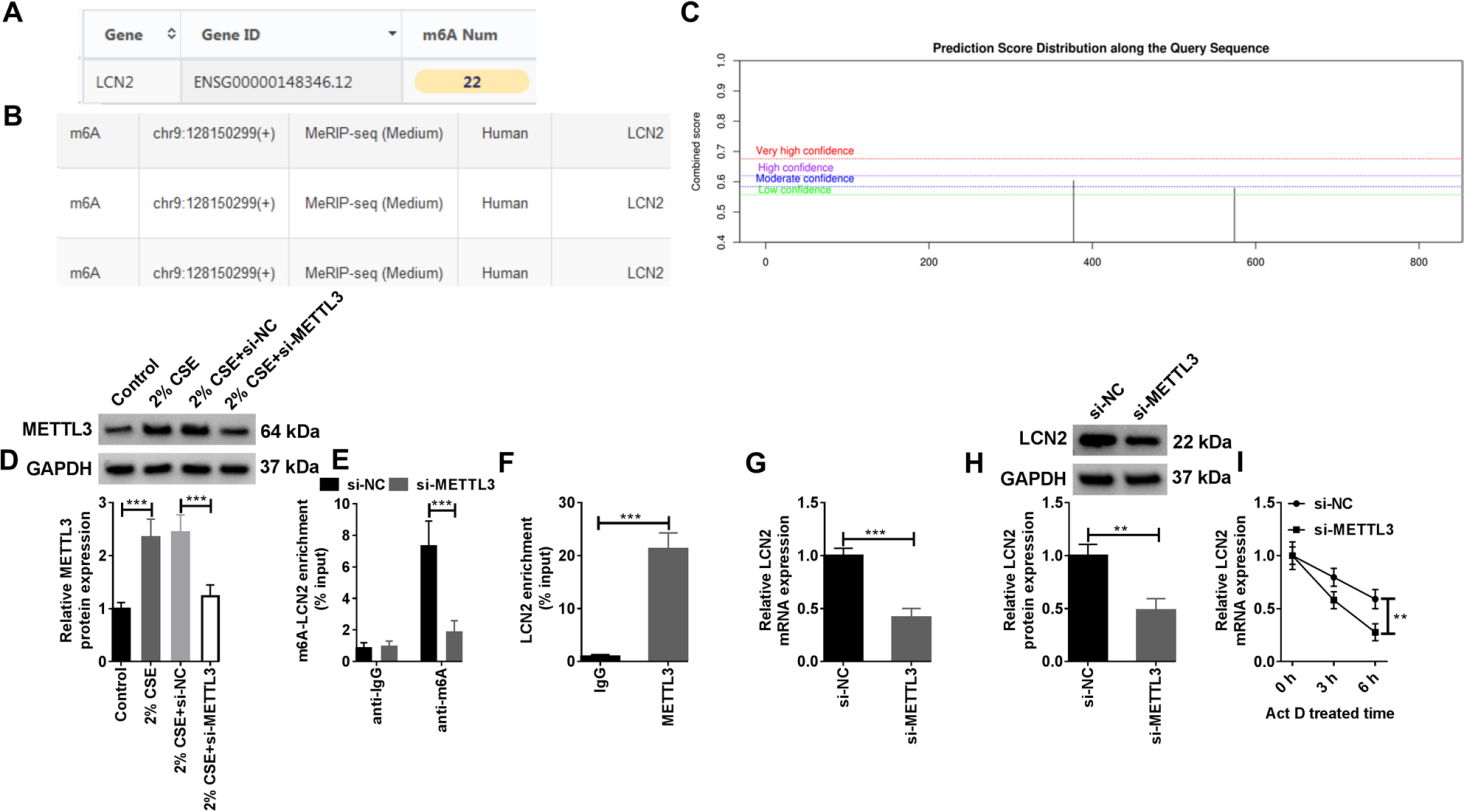
METTL3 mediates the m6A methylation of LCN2. **A **and **B** The presence of m6A methylation modification in LCN2 was predicted by the RMbase and RMvar databases. Statistical analysis of the data was performed using one-way and two-way ANOVA with Turkey’s post hoc test. **C** The presence of LCN2 methylation modification sites was predicted by the SRAMP website. Statistical analysis of the data was performed using one-way ANOVA with Turkey’s post hoc test. (D-I) HMVECs were transfected with si-NC and si-METTL3. **D** WB analysis of METTL3 protein levels in HMVECs (Biological replicates, *N* = 3). Statistical analysis of the data was performed using two-way ANOVA with Turkey’’s post hoc test. **E** The m6A methylation level of LCN2 in HMVECs was determined by MeRIP assay (Biological replicates, *N* = 3). **F** RIP assay was used to verify the interaction between METTL3 and LCN2 (Biological replicates, *N* = 3). **G **and **H** Changes in LCN2 mRNA and protein levels after knockdown of METTL3 were determined by RT-qPCR and WB (Biological replicates, *N* = 3). **I** HMVECs with METTL3 knockdown were treated with actinomycin D and the expression level of LCN2 mRNA was detected by RT-qPCR (Biological replicates, *N* = 3). Statistical analysis of the data was performed using Student’s *t*-test. **P < 0.01, ***P < 0.001

To clarify the role of METTL3 in the regulation of LCN2, bioinformatics analysis was performed. It was predicted that there was m6A methylation on the mRNA sequence of LCN2 through RMbase and RMvar databases, and SRAMP website confirmed the existence of methylation modification sites on LCN2 (Fig. 2A-C). WB analysis displayed that CSE could also upregulate the level of METTL3 in HMVECs, and the protein level of METTL3 was greatly impeded after transfection with si-METTL3 (Fig. 2D). When METTL3 was silenced, the level of m6A methylation modification of LCN2 in HMVECs was also curbed (Fig. 2E). In contrast, when METTL3 was overexpressed, METTL3 protein level was upregulated and the level of m6A modification of LCN2 was elevated (Supplementary Fig. 1 A and B). At the same time, RIP assay confirmed that METTL3 was able to bind to LCN2 mRNA (Fig. 2F). It is worth mentioning that when METTL3 was silenced, the protein and mRNA levels of LCN2 were similarly repressed in HMVEC (Fig. 2G and H). Moreover, using actinomycin D treatment of HMVECs, it was observed that when METTL3 was silenced, the mRNA stability of LCN2 was also reduced (Fig. 2I). Furthermore, to further determine the targeted regulation of METTL3 on LCN2, we knocked down the m6A methyltransferases METTL14, METTL16, FTO and the 5-methylcytosine (m5C) methyltransferases NSUN2, and the changes of LCN2 mRNA level were detected by RT-qPCR. The results were shown in Supplementary Fig. 2. It was found that knockdown of METTL14 resulted in a change in the mRNA level of LCN2, but the magnitude of the change was much smaller than that of METTL3 knockdown. Besides, knockdown of METTL16, FTO and NSUN2 had no significant effect on LCN2 mRNA level. In a word, METTL3 stabilized LCN2 expression by m6A methylation modification in HMVECs.

### IGF2BP3 mediated the m6A methylation of LCN2

**Fig. 3:**
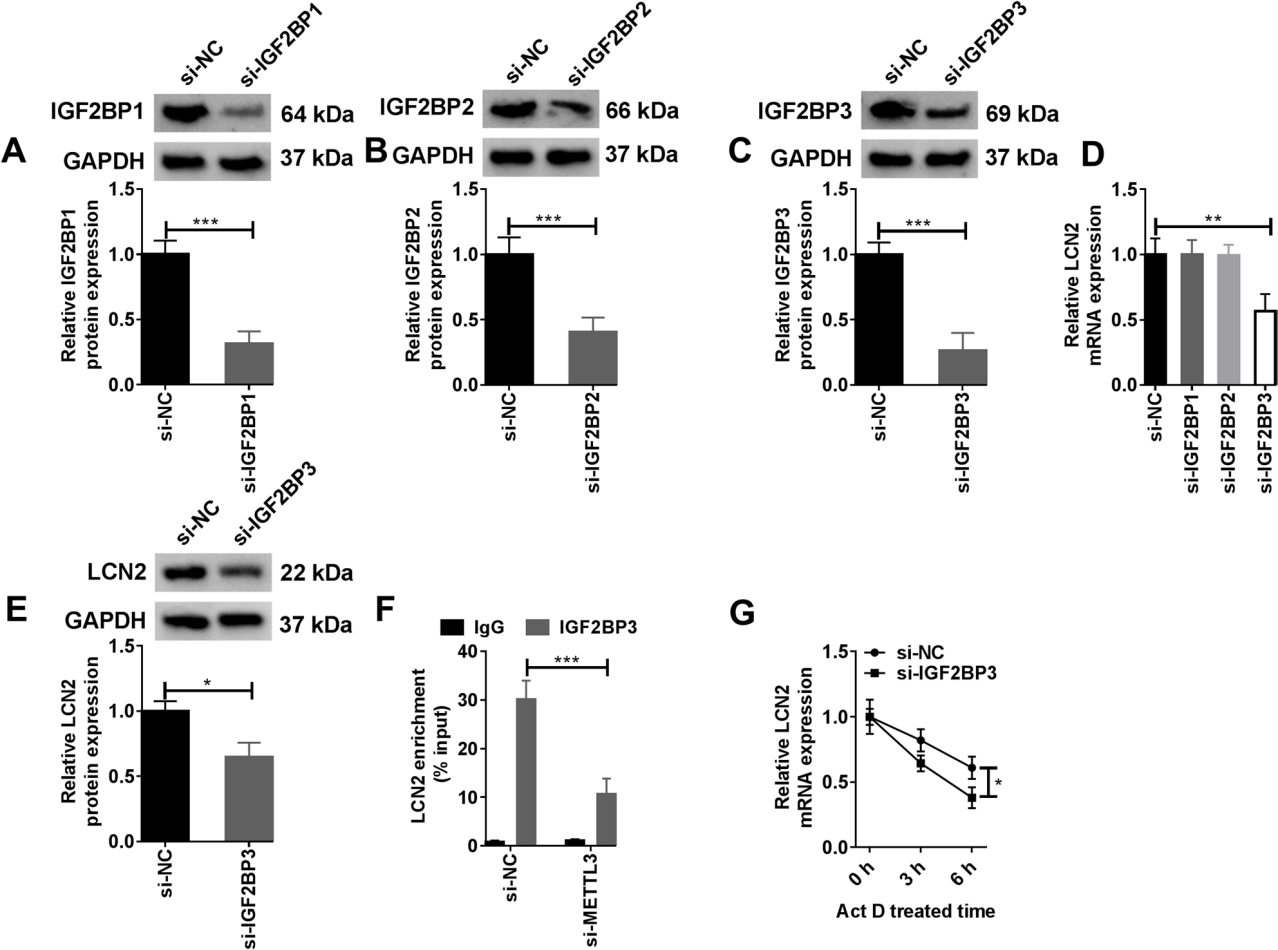
IGF2BP3 mediates the m6A methylation of LCN2. **A**-**G** HMVECs were transfected with si-NC, si-IGF2BP1, si-IGF2BP2, si-IGF2BP3, and si-METTL3. **A**-**C** WB was used to detect the expression of IGF2BP1, IGF2BP2, and IGF2BP3 proteins in HMVECs transfected with si-IGF2BP1, si-IGF2BP2, and si-IGF2BP3 (Biological replicates, *N* = 3). Statistical analysis of the data was performed using Student’s *t*-test. **D** The mRNA expression levels of LCN2 in HMVECs transfected with si-IGF2BP1, si-IGF2BP2, and si-IGF2BP3 were detected by RT-qPCR (Biological replicates, *N* = 3). Statistical analysis of the data was performed using one-way ANOVA with Turkey’s post hoc test. **E** WB was used to detect the protein expression of LCN2 in HMVECs transfected with si-IGF2BP3 (Biological replicates, *N* = 3). Statistical analysis of the data was performed using Student’s *t*-test. **F** RIP assay was utilized to verify the binding of IGF2BP3 to LCN2 (Biological replicates, *N* = 4). Statistical analysis of the data was performed using two-way ANOVA with Turkey’’s post hoc test. **G** HMVECs with IGF2BP3 knockdown were treated with actinomycin D and the expression level of LCN2 mRNA was detected by RT-qPCR (Biological replicates, *N* = 3). Statistical analysis of the data was performed using Student’s *t*-test. *P < 0.05, **P < 0.01, ***P < 0.001

IGF2BP1, IGF2BP2, and IGF2BP3 were introduced to further clarify the specific mechanism of METTL3-mediated LCN2 m6A methylation. After transfection of si-IGF2BP1, si-IGF2BP2 and si-IGF2BP3 into HMVECs, WB results showed that the protein levels of IGF2BP1, IGF2BP2 and IGF2BP3 were remarkably inhibited (Fig. 3A-C). When the expression of LCN2 was examined, it was found that the LCN2 mRNA level was reduced only when IGF2BP3 was silenced (Fig. 3D). Subsequently, WB analysis confirmed that knockdown of IGF2BP3 could block the protein expression of LCN2 in HMVECs (Fig. 3E). When YTHDF1, YTHDF2, YTHDF3 and YTHDC1 were knocked down, the RT-qPCR results showed that the above reading proteins had no significant effect on the mRNA level of LCN2 (Supplementary Fig. 3). It was confirmed that IGF2BP3 targeted LCN2. More importantly, the binding of IGF2BP3 to LCN2 was demonstrated by RIP assay (Fig. 3F). Similar to the effect of METTL3 knockdown, silencing IGF2BP3 also reduced the stability of LCN2 mRNA (Fig. 3G). In short, IGF2BP3 could bind to the LCN2 mRNA and induce its m6A methylation modification, thereby stabilizing the level of LCN2 in HMVECs.

### METTL3 inhibitor STM2457 rescued CSE induced HMVEC injury and ferroptosis

**Fig. 4:**
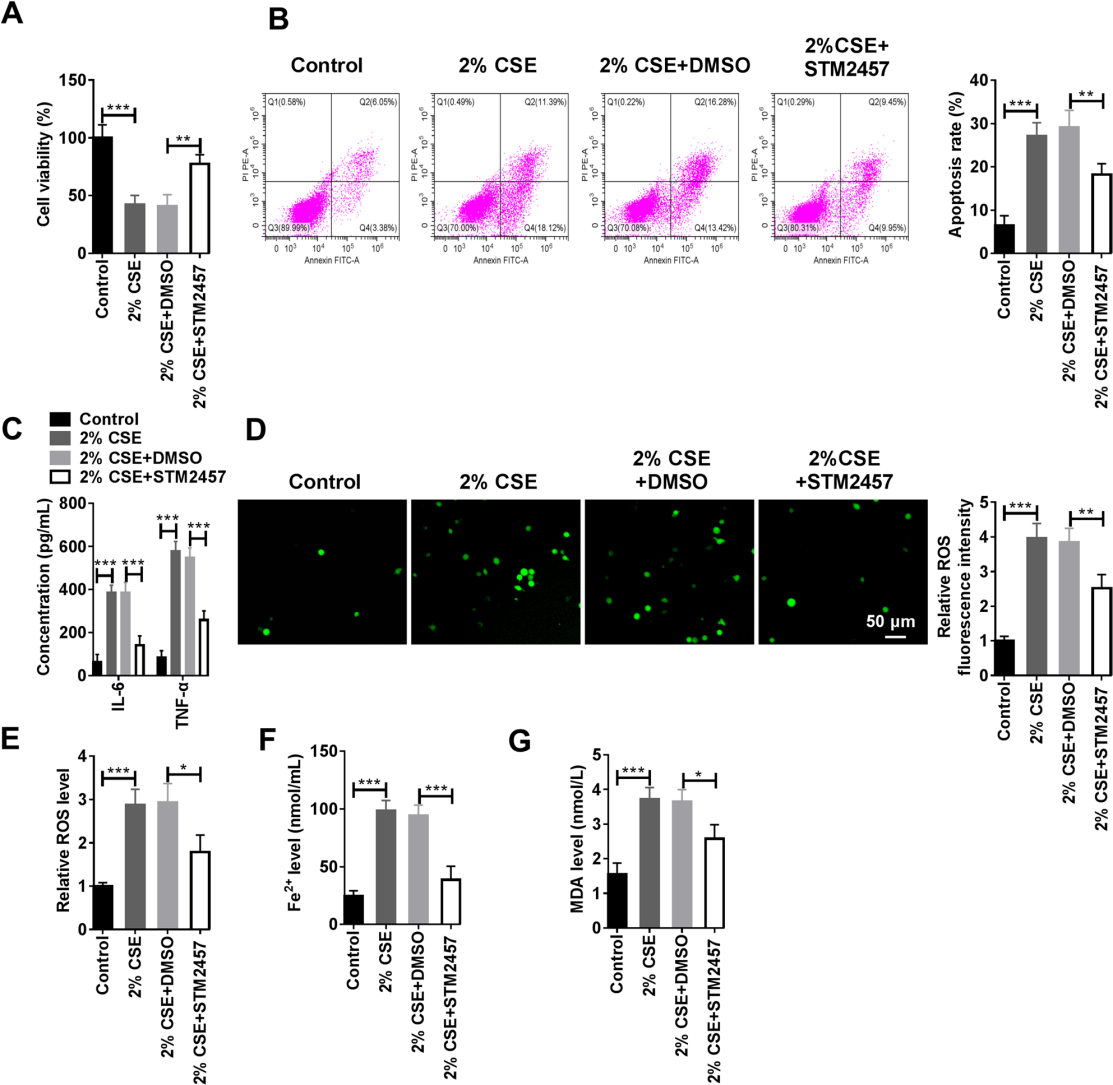
Effect of METTL3 inhibitor STM2457 on CSE induced HMVEC injury and ferroptosis. **A**-**J** HMVECs were divided into the control group, 2% CSE group, 2% CSE + DMSO group, and 2% CSE + STM2457 group. **A** The viability of HMVECs was monitored by CCK-8 experiment (Biological replicates, *N* = 3). **B** The apoptosis of HMVECs were monitored by flow cytometry (Biological replicates, *N* = 3). **C** The levels of IL-6 and TNF-α were measured by ELISA kits (Biological replicates, *N* = 3). **D **and **E** ROS levels in HMVECs were measured by immunofluorescence and flow cytometry (Biological replicates, *N* = 3). **F **and **G** Fe^2+^ and MDA levels in HMVECs were measured by the corresponding kits (Biological replicates, *N* = 3). Statistical analysis of the data was performed using one-way and two-way ANOVA with Turkey’s post hoc test. *P < 0.05, **P < 0.01, ***P < 0.001

DMSO was used as the control for STM2457 to observe the effect of STM2457 on CSE-induced HMVECs. CCK-8 assay result revealed that STM2457 effectively restored the activity of HMVECs, which were compromised following exposure to CSE (Fig. 4A). And STM2457 could also reduce the apoptosis rate of HMVECs increased by CSE (Fig. 4B). Similarly, the levels of IL-6 and TNF-α, which were upregulated by CSE, were decreased with the addition of STM2457 (Fig. 4C). What’s more, high levels of ROS, Fe^2+^, and MDA in CSE-exposed HMVECs were conspicuously reduced when STM2457 was present (Fig. 4D-G). In conclusion, METTL3 inhibitor STM2457 could effectively alleviated cell injury and ferroptosis in HMVECs exposed to CSE.

### Overexpression of LCN2 destroyed the effect of METTL3 Silencing on CSE-induced HMVECs damage

**Fig. 5:**
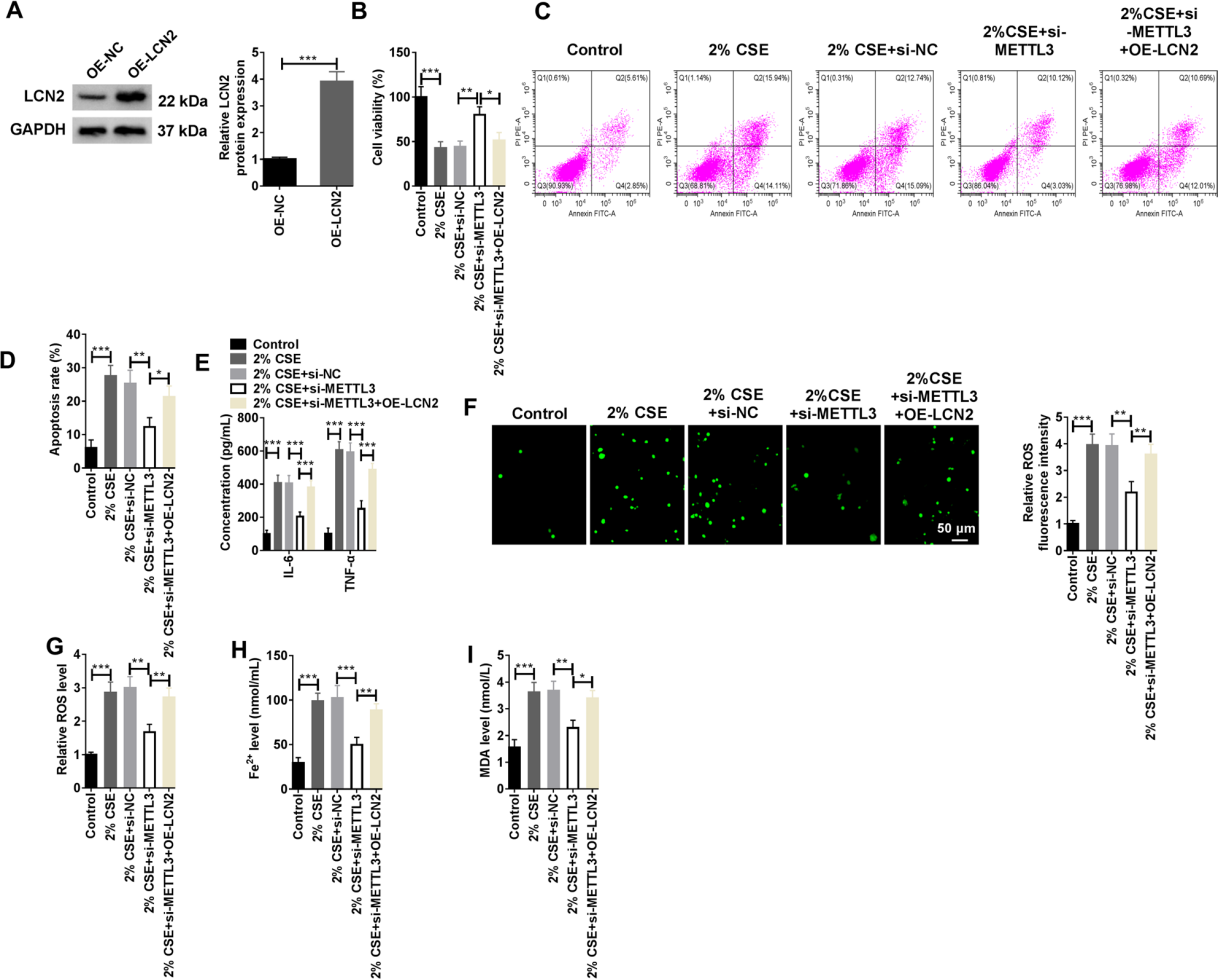
Effect of overexpression of LCN2 after silencing METTL3 on CSE-induced HMVEC injury. **A** The overexpression efficiency of LCN2 was determined by WB after HMVEC was transfected with OE-LCN2 (Biological replicates, *N* = 3). **B**-**I** HMVECs were divided into the control group, 2% CSE group, 2% CSE + si-NC group, 2% CSE + si-METTL3 group, and 2% CSE + si-METTL3 + OE-LCN2 group. **B** CCK-8 experiments was employed to detect the viability of HMVECs (Biological replicates, *N* = 3). **C **and **D** Flow cytometry were applied to detect the apoptosis of HMVECs (Biological replicates, *N* = 3). **E** The levels of IL-6 and TNF-α were measured by ELISA kits (Biological replicates, *N* = 3). **F **and **G** ROS levels in HMVECs were measured by immunofluorescence and flow cytometry (Biological replicates, *N* = 3). **H **and **I** Fe^2+^ and MDA levels in HMVECs were measured by the corresponding kits. Fe^2+^ and MDA levels in HMVECs were measured by the corresponding kits (Biological replicates, *N* = 3). Statistical analysis of the data was performed using one-way and two-way ANOVA with Turkey’s post hoc test. *P < 0.05, **P < 0.01, ***P < 0.001

LCN2 was overexpressed in the presence of METTL3 silencing to clear the role of METTL3/LCN2 in CSE-induced HMVECs injury. When HMVECs were transfected with OE-LCN2, the protein level of LCN2 was prominently upregulated (Fig. 5A). As shown in Fig. 5B, knockdown of METTL3 was able to restore the inhibition of CSE on cell viability of HMVECs, but when LCN2 was overexpressed, cell viability was re-impaired. Conversely, when METTL3 was silenced, the apoptosis rate of HMVECs was also decreased, but this effect was again abolished by overexpression of LCN2 (Fig. 5C and D). In addition, the levels of IL-6, TNF-α, ROS, Fe^2+^, and MDA were markedly decreased following the silencing of METTL3 (Fig. 5E-I). Moreover, si-METTL3 also impeded the upregulation of PTGS2 in response to CSE exposure. At the same time, the GSH level decreased by CSE increased again (Supplementary Fig. 4 A and B). Nevertheless, the overexpression of LCN2 was capable of restoring their levels to previous states. In general, both METTL3 and LCN2 were involved in CSE-induced HMVECs injury.

### METTL3 regulated the NRF2/SLC7A11/GPX4 pathway by stabilizing LCN2

**Fig. 6:**
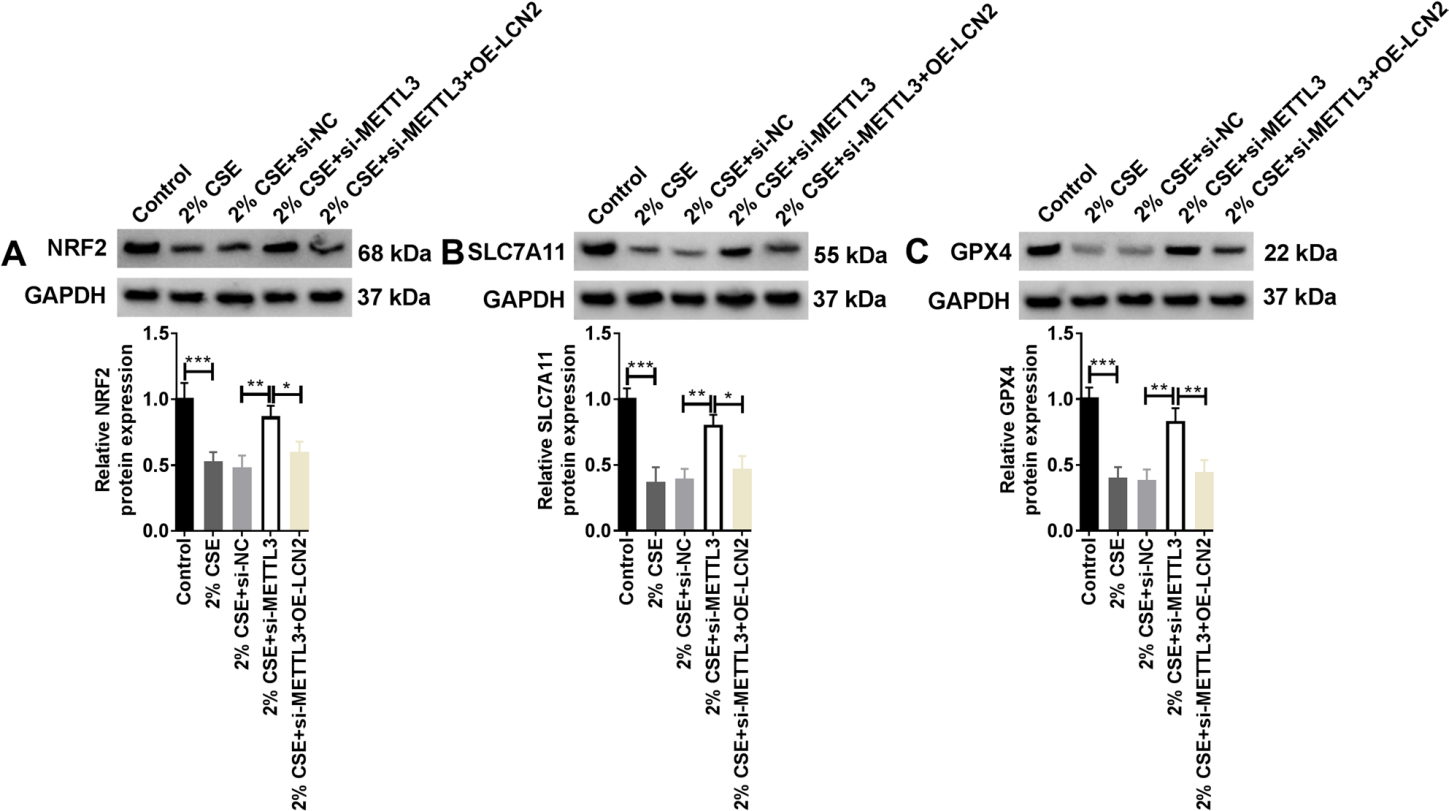
Effect of overexpression of LCN2 after silencing METTL3 on the NRF2/SLC7A11/GPX pathway. HMVECs were divided into the control group, 2% CSE group, 2% CSE + si-NC group, 2% CSE + si-METTL3 group, and 2% CSE + si-METTL3 + OE-LCN2 group. **A**-**C** Protein levels of NRF2, SLC7A11, and GPX4 in HMVECs were monitored by WB (Biological replicates, *N* = 3). Statistical analysis of the data was performed using one-way ANOVA with Turkey’s post hoc test. *P < 0.05, **P < 0.01, ***P < 0.001

Analysis of the NRF2/SLC7A11/GPX4 pathway related proteins by WB presented that the protein levels of Nuclear factor E2-related factor 2 (NRF2), Solute carrier family 7 member 11 (SLC7A11) and Glutathione peroxidase 4 (GPX4) were conspicuously downregulated in HMVECs exposed to CSE. When the expression of METTL3 was constrained, the expression of the above three showed an upward trend. However, when LCN2 was overexpressed, the effect of METTL3 knockdown was completely destroyed (Fig. 6A-C). These results suggested that METTL3/LCN2 axis aggravated CSE damage to HMVECs by impeding NRF2/SLC7A11/GPX4 pathway.

### Knockdown of LCN2 could alleviate lung injury in COPD mice

**Fig. 7:**
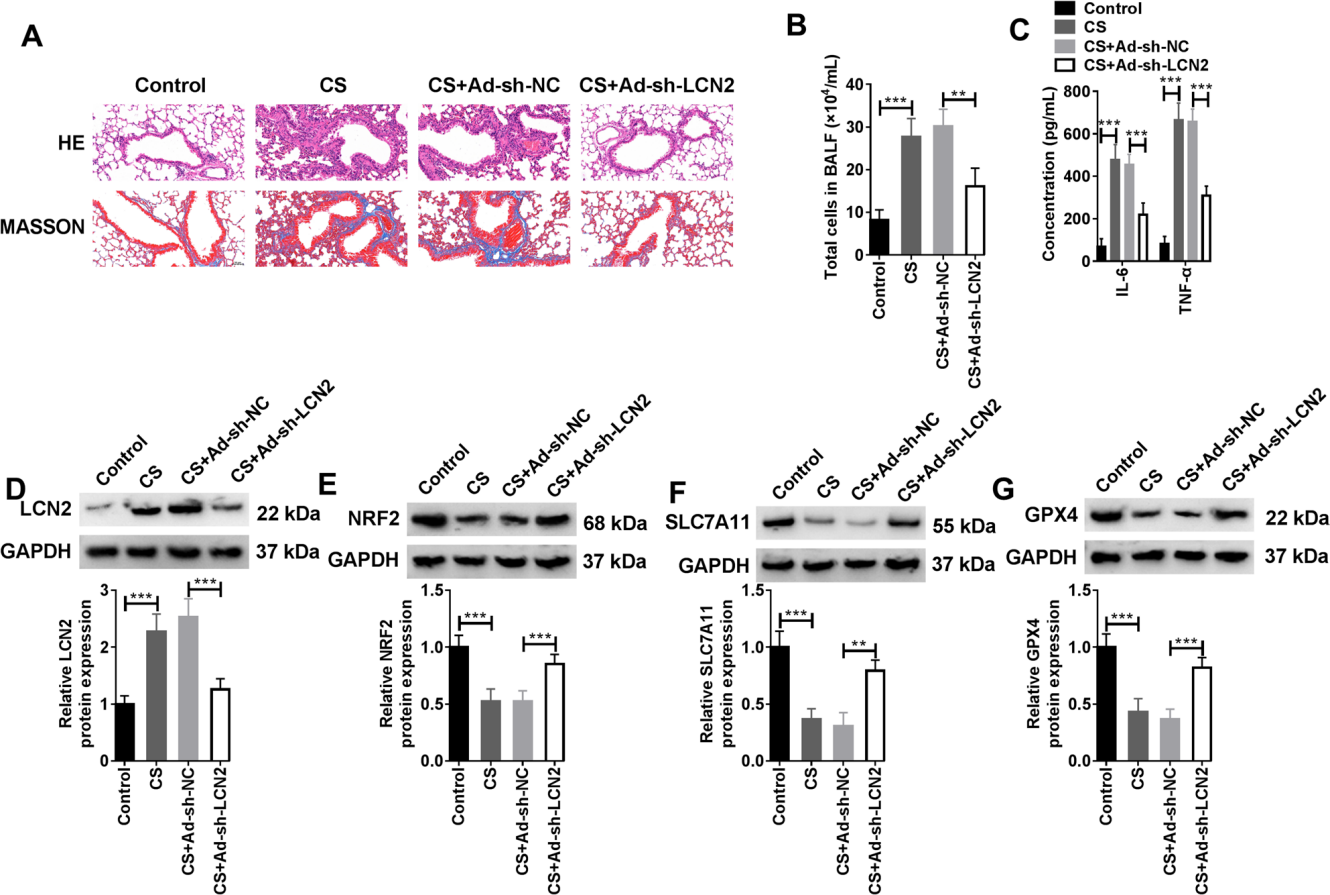
**Effect of silencing LCN2 in an** in vivo **model of COPD.** Mice were divided into 4 groups: the Control group, the CS group, the CS + Ad-sh-NC group, and the CS + Ad-sh-LCN2 group (*n* = 8). **A** Results of HE and MASSON staining of mouse lung tissues (Scale bar: 50 μm). **B** Cell counting results in BALF of mice in each group (Biological replicates, *N* = 3). **C** The corresponding kits were used to detect IL-6 and TNF-α secretion (Biological replicates, *N* = 3). **D**-**E** The protein levels of LCN2, NRF2, SLC7A11, and GPX4 in lung tissues of mice in each group were monitored by WB (Biological replicates, *N* = 3). Statistical analysis of the data was performed using one-way and two-way ANOVA with Turkey’s post hoc test. **P < 0.01, ***P < 0.001

To further explore the role of LCN2 in vivo, a model of COPD was constructed. HE staining of lung tissue showed that the CS exposure group had disordered alveolar structure, thinner alveolar septum, enlarged alveolar cavity, and more pulmonary bulla, and the average number of alveoli was reduced. MASSON staining presented obvious fibrosis in the pulmonary interstitium and peribronchial tissue, obvious thickening of the bronchial epithelium, and fascicular deposition of collagen fibers in the lung tissue. However, the above pathological changes were significantly alleviated in CS + Ad-sh-LCN2 group (Fig. 7A). The number of cells in BALF of CS group increased, and the number of cells in BALF of CS + Ad-sh-NC group decreased significantly (Fig. 7B). At the same time, the levels of inflammatory factors IL-6 and TNF-α, which were upregulated by CS-exposure, were also effectively reduced by LCN-2 knockdown (Fig. 7C). The protein level of LCN2 was increased in CS group and decreased in CS + Ad-sh-LCN2 group by WB (Fig. 7D). The protein levels of NRF2, SLC7A11 and GPX4 were upregulated by CS, while LCN2 knockdown completely abolished the inhibitory effect of CS on their protein levels (Fig. 7E-G). VEGFA is a key signaling protein that supports tissue growth and repair primarily by promoting the formation of new blood vessels. The protein level of VEGFA was down-regulated after CS exposure. When LCN2 expression was inhibited, the level of VEGFA displayed an upward trend (Supplementary Fig. 5). In summary, in vivo results demonstrated that overexpression of LCN2 caused lung injury in COPD mice and curbed the activation of NRF2/SLC7A11/GPX4 signaling pathway.

## Discussion

COPD is often associated with various long-term factors, including smoking habits, air pollution, exposure to dust in the occupational environment, and frequent respiratory infections [33]. Its core pathological feature is a persistent inflammatory response, characterized by the infiltration of numerous inflammatory cells in the airways and parenchymal regions of the lungs. This chronic inflammatory stimulation can prompt structural changes in the airways, leading to emphysema and impaired lung function [34, 35]. Furthermore, the oxidative stress response in COPD patients is significantly enhanced, posing a threat to lung tissue cells and leading to issues such as DNA structural damage, abnormal protein function, and lipid peroxidation [36]. These problems further exacerbate the inflammatory response and worsen airway damage [37]. Currently, the primary focus of COPD treatment strategies is on symptom relief, enhancing patients’ quality of life, and preventing disease progression. However, these existing treatment methods are often accompanied by significant side effects and cannot achieve a cure for the disease. Additionally, for some severely ill patients, surgical treatment carries high risks and is expensive [38, 39]. In light of these limitations of current treatment technologies, the search for biomarkers for targeted therapy offers new hope and possibilities for the treatment of COPD. In the study by Wang et al., CSE treatment upregulated LCN2 expression in HBECs in a concentration-dependent manner and could induce apoptosis and ferroptosis in HBECs [21]. In this study, we observed similar results. Specifically, when HMVECs were exposed to CSE, there was a significant reduction in cell viability, accompanied by an increase in apoptosis rate, elevated secretion of inflammatory cytokines, and promotion of ferroptosis. However, once the expression of LCN2 was inhibited, these adverse effects on HMVECs could be effectively reversed. Therefore, it can be concluded that high levels of LCN2 not only amplify COPD airway inflammation by releasing inflammatory factors, but also link inflammation and ferroptosis through an “iron-dependent pathway”. Next, we will delve into the precise molecular mechanisms by which LCN2 exacerbates COPD.

The emergence and progression of diseases are intricately linked to epigenetic abnormalities, and m6A methylation modification represents a ubiquitous and profoundly significant epigenetic alteration [40]. Through bioinformatics analysis, specific sites of m6A methylation modification within the mRNA sequence of LCN2 were predicted. It was reported that the m6A methyltransferase METTL3 exhibited upregulated expression in COPD patients and cellular models induced by smoking, and it regulated the expression of suppressor of cytokine signaling 3 (SOCS3) through m6A methylation modification, which facilitated the activation of the signal transducer and activator of transcription 3 (STAT3)/snail family transcriptional repressor 1 (SNAI1) signaling and the process of epithelial-mesenchymal transition (EMT) [41]. Another study showed that maternally expressed gene 3 (MEG3) upregulated the m6A methylation level of triggering receptor expressed on myeloid cells 1 (TREM-1) through the recruitment of Spi-1 proto-oncogene (SPI1), which in turn activated METTL3. Overexpression of either TREM-1 or METTL3 negated the protective benefits of MEG3 inhibitors on M1 polarization, pyroptosis, and lung injury in vivo [42]. Therefore, with the help of MeRIP and RIP techniques, it was confirmed that METTL3 could mediate m6A methylation of LCN2 and ensure the stable expression of LCN2 mRNA. But how does METTL3 mediate m6A methylation of LCN2? It is commonly recognized that m6A methylation modification undergoes a reversible process regulated by methylases, demethylases and methylation reading proteins [43, 44]. The m6A methylation modification regulates gene expression by affecting the structure and function of RNA. Methylation-reading proteins recognize and bind to these modified RNA sequences to further regulate the expression of downstream genes [45]. We introduced the Insulin-like growth factor 2 mRNA-binding protein (IGF2BP) that uses its distinctive RNA binding domain (RBD) and its flanking regions to identify and interact with m6A modified RNAs. Unlike the known YTH domain proteins, IGF2BP does not promote mRNA degradation, but rather enhances mRNA stability and translational efficiency [46, 47]. IGF2BP3 was verified to bind to LCN2 and regulate its mRNA stability. The conclusion drawn was that METTL3 served as the mediator for the m6A methylation of LCN2, and the recognition of IGF2BP3 to the m6A methylation site further enhanced the stability of LCN2 mRNA.

Based on this, we aimed to further investigate whether this regulatory mechanism had an impact on the progression of COPD. STM2457 was confirmed to have a direct binding affinity for the SAM binding site of METTL3, effectively retarding the methyltransferase activity [48]. In the current study, STM2457 could effectively alleviate the adverse effects of CSE exposure on HMVECs, and was indirectly confirmed that METTL3 was involved in the progression of COPD. Subsequently, LCN2 was overexpressed when METTL3 was silenced for reversion experiments. It was observed that the effects of silencing METTL3 on the viability, proliferation, apoptosis, the release of inflammatory factors, and ferroptosis of HMVECs induced by CSE were completely abolished by overexpression of LCN2, revealing that METTL3/IGF2BP3 mediated m6A methylation modification of LCN2 to cause HMVEC damage and stimulate ferroptosis in COPD. Notably, one study indicated that m6A-modified circSAV1 could promote the translation of iron responsive element binding protein 2 (IREB2) mRNA by recruiting YTHDF1, and high levels of IREB2 could induce the accumulation of lipid peroxides and trigger ferrodeath in lung epithelial cells, further aggravating the progression of COPD [49]. Moreover, Lu et al. demonstrated that the IGF2BP3-NRF2 axis is able to regulate ferroptosis in hepatocellular carcinoma [50]. Likewise, another report illustrated that the m6A reader protein IGF2BP3 induced ferroptosis in glioma by regulating GPX4 expression [51]. Based on this, we hypothesized that METTL3/IGF2BP3 may be involved in the malignant development of COPD by mediating m6A methylation modification of LCN2. By carrying out the recovery experiment with overexpression of LCN2 on the basis of METTL3 knockdown, it could be seen that overexpression of LCN2 could completely eliminate the beneficial effect of METTL3 knockdown on CSE-exposed HMVECs. It was implicated that METTL3 induced m6A methylation modification of LCN2 and recruited IGF2BP3 to recognize m6A modification sites on LCN2 mRNA, thereby suppressing the growth of HMVECs in COPD, facilitating the inflammatory response and ferroptosis. These effects further aggravate the condition of COPD. Ferroptosis primarily occurs through the peroxidation of unsaturated fatty acids abundant on the cell membrane, driven by the action of divalent iron or lipoxygenases, ultimately leading to cell death [52]. Furthermore, ferroptosis was also evident through a reduction in GPX4, a pivotal regulatory enzyme within the antioxidant system. Nuclear factor erythroid 2-related factor 2 (NRF2) is an important regulator of antioxidant stress [53]. Upon exposure to oxidative stress, NRF2 moves from the cytoplasm to the nucleus, where it interacts with antioxidant response elements (AREs), subsequently initiating the transcription and expression of various antioxidant genes [54]. When NRF2 is activated, it can increase solute carrier family 7 member 11 (SLC7A11) expression, thereby promoting cysteine transport and GSH synthesis, which in turn enhances glutathione peroxidase 4 (GPX4) activity and protects cells from ferroptosis damage. However, when SLC7A11 expression is reduced, cysteine transport is blocked, which in turn affects glutathione (GSH) synthesis and GPX4 activity. Decreased activity or decreased expression of GPX4 increases the sensitivity of cells to ferroptosis [55–57]. Upon monitoring the levels of related proteins, we found that knocking down METTL3 could reverse the downregulation of NRF2, SLC7A11, and GPX4 protein levels that was caused by CSE, thereby reducing the sensitivity of HMVECs to ferroptosis. However, when LCN2 was overexpressed, the aforementioned positive effects brought about by knocking down METTL3 completely disappeared, and HMVECs once again became susceptible to ferroptosis and suffered corresponding damage. Most crucially, it was discovered that inhibiting LCN2 expression in the in vivo COPD model could effectively mitigate the lung injury and inflammatory response induced by CS in mice. Simultaneously, this inhibition also promoted the activation of the NRF2/SLC7A11/GPX4 pathway.

Despite this, the current work still exhibits certain deficiencies. On the one hand, this study merely confirmed the function of LCN2 in a mouse model of COPD at the animal level, without delving into whether the m6A regulatory mechanism exerted by METTL3/IGF2BP3 on LCN2 is linked to the in vivo progression of COPD. On the other hand, COPD is a complex disease involving the airway, lung parenchyma and pulmonary blood vessels. In this study, only a single cell line of HMVECs was used, which could not simulate the interaction network between different cells in the pathological state of COPD. Meanwhile, the function of endothelial cells in different regions of the lung tissue of COPD patients is heterogeneous, and a single cell line cannot cover this regional specific difference, which may lead to the limitation of the study results. Moreover, as this study has not been validated with clinical samples, it is challenging to ascertain the precise correlation between the study’s conclusions and the pathological processes in COPD patients.

Taken together, this study elucidated that METTL3/IGF2BP3 negatively affected HMVECs growth by mediating m6A methylation modification of LCN2, while inducing the progression of ferroptosis via the NRF2/SLC7A11/GPX4 pathway in COPD. This study fills the gap in the current research on the regulation of ferroptosis by m6A in COPD. It also provides a new direction and theoretical basis for the development of therapeutic strategies for COPD targeting m6A modification or NRF2 pathway.

## Supplementary Information

Supplementary Material 1.

Supplementary Material 2.

Supplementary Material 3.

Supplementary Material 4.

Supplementary Material 5.

Supplementary Material 6.

## Data Availability

The data are available from the corresponding author on reasonable request.

## References

[1] López-Campos JL, Tan W, Soriano JB. Global burden of COPD. Respirology. 2016;21:14–23. 26494423 10.1111/resp.12660

[2] Xu J, Zeng Q, Li S, Su Q, Fan H. Inflammation mechanism and research progress of COPD. Front Immunol. 2024;15:1404615. 39185405 10.3389/fimmu.2024.1404615 PMC11341368

[3] Bagdonas E, Raudoniute J, Bruzauskaite I, Aldonyte R. Novel aspects of pathogenesis and regeneration mechanisms in COPD. Int J Chron Obstruct Pulmon Dis. 2015;10:995–1013. 26082624 10.2147/COPD.S82518 PMC4459624

[4] Siafakas NM, Antoniou KM, Tzortzaki EG. Role of angiogenesis and vascular remodeling in chronic obstructive pulmonary disease. Int J Chron Obstruct Pulmon Dis. 2007;2:453–62. 18268919 PMC2699970

[5] Borek I, Birnhuber A, Voelkel NF, Marsh LM, Kwapiszewska G. The vascular perspective on acute and chronic lung disease. J Clin Invest. 2023;133:e170502. 37581311 10.1172/JCI170502 PMC10425217

[6] Meng D, Zhu C, Jia R, Li Z, Wang W, Song S. The molecular mechanism of ferroptosis and its role in COPD. Front Med (Lausanne). 2022;9:1052540. 36687445 10.3389/fmed.2022.1052540 PMC9852995

[7] Xu W, Deng H, Hu S, Zhang Y, Zheng L, Liu M, et al. Role of ferroptosis in lung diseases. J Inflamm Res. 2021;14:2079–90. 34045882 10.2147/JIR.S307081 PMC8144020

[8] Li J, Cao F, Yin HL, Huang ZJ, Lin ZT, Mao N, et al. Ferroptosis: past, present and future. Cell Death Dis. 2020;11:88. 32015325 10.1038/s41419-020-2298-2 PMC6997353

[9] Mao H, Zhao Y, Li H, Lei L. Ferroptosis as an emerging target in inflammatory diseases. Prog Biophys Mol Biol. 2020;155:20–8. 32311424 10.1016/j.pbiomolbio.2020.04.001

[10] Yu S, Jia J, Zheng J, Zhou Y, Jia D, Wang J. Recent progress of ferroptosis in lung diseases. Front Cell Dev Biol. 2021;9:789517. 34869391 10.3389/fcell.2021.789517 PMC8635032

[11] Oerum S, Meynier V, Catala M, Tisné C. A comprehensive review of m6A/m6Am RNA methyltransferase structures. Nucleic Acids Res. 2021;49:7239–55. 34023900 10.1093/nar/gkab378 PMC8287941

[12] He PC, He C. m(6) A RNA methylation: from mechanisms to therapeutic potential. EMBO J. 2021;40:e105977. 33470439 10.15252/embj.2020105977 PMC7849164

[13] An Y, Duan H. The role of m6A RNA methylation in cancer metabolism. Mol Cancer. 2022;21:14. 35022030 10.1186/s12943-022-01500-4 PMC8753874

[14] Widagdo J, Anggono V. The m6A-epitranscriptomic signature in neurobiology: from neurodevelopment to brain plasticity. J Neurochem. 2018;147:137–52. 29873074 10.1111/jnc.14481

[15] Xu Y, Liu W, Ren L. Role of m6A RNA methylation in ischemic stroke. Mol Neurobiol. 2024;61:6997–7008. 38363537 10.1007/s12035-024-04029-3

[16] Huang X, Lv D, Yang X, Li M, Zhang H. m6A RNA methylation regulators could contribute to the occurrence of chronic obstructive pulmonary disease. J Cell Mol Med. 2020;24:12706–15. 32961012 10.1111/jcmm.15848 PMC7686997

[17] Guo X, Lin Y, Lin Y, Zhong Y, Yu H, Huang Y, et al. PM2.5 induces pulmonary microvascular injury in COPD via METTL16-mediated m6A modification. Environ Pollut. 2022;303:119115. 35259473 10.1016/j.envpol.2022.119115

[18] Xie B, Dai Z, Jiang C, Gao X, Yang S, Peng M, et al. ZC3H13 promotes ITGA6 m(6)A modification for chronic obstructive pulmonary disease progression. Cell Signal. 2024;120:111190. 38670474 10.1016/j.cellsig.2024.111190

[19] Liu S, Zhuo L, Wang J, Zhang Q, Li Q, Li G, et al. METTL3 plays multiple functions in biological processes. Am J Cancer Res. 2020;10:1631–46. 32642280 PMC7339281

[20] Song B, Zeng Y, Cao Y, Zhang J, Xu C, Pan Y, et al. Emerging role of METTL3 in inflammatory diseases: mechanisms and therapeutic applications. Front Immunol. 2023;14:1221609. 37671161 10.3389/fimmu.2023.1221609 PMC10475571

[21] Wang R, Xu J, Wei S, Liu X. Increased Lipocalin 2 detected by RNA sequencing regulates apoptosis and ferroptosis in COPD. BMC Pulm Med. 2024;24:535. 39462322 10.1186/s12890-024-03357-3 PMC11515215

[22] Xiao X, Yeoh BS, Vijay-Kumar M. Lipocalin 2: an emerging player in iron homeostasis and inflammation. Annu Rev Nutr. 2017;37:103–30. 28628361 10.1146/annurev-nutr-071816-064559

[23] Tang W, Zhai R, Ma J, Xu G. Lipocalin-2-mediated ferroptosis as a target for protection against light-induced photoreceptor degeneration. Mol Med. 2025;31:190. 40375133 10.1186/s10020-025-01250-1 PMC12083120

[24] Pan Z, Li B, Lu P, Rong G, Wang X. Inhibiting LCN2 can suppress the development of NSCLC by promoting ferroptosis. Gene. 2024;894:148026. 38000702 10.1016/j.gene.2023.148026

[25] Tan M, He Y, Yi J, Chen J, Guo Q, Liao N, et al. WTAP mediates NUPR1 regulation of LCN2 through m(6)A modification to influence Ferroptosis, thereby promoting breast cancer Proliferation, migration and invasion. Biochem Genet. 2024;62:876–91. 37477758 10.1007/s10528-023-10423-8

[26] Treekitkarnmongkol W, Hassane M, Sinjab A, Chang K, Hara K, Rahal Z, et al. Augmented Lipocalin-2 is associated with chronic obstructive pulmonary disease and counteracts lung adenocarcinoma development. Am J Respir Crit Care Med. 2021;203:90–101. 32730093 10.1164/rccm.202004-1079OC PMC7781147

[27] Alter P, Baker JR, Dauletbaev N, Donnelly LE, Pistenmaa C, Schmeck B, et al. Update in chronic obstructive pulmonary disease 2019. Am J Respir Crit Care Med. 2020;202:348–55. 32407642 10.1164/rccm.202002-0370UP PMC8054880

[28] Lee KH, Woo J, Kim JY, Lee CH, Yoo CG. Cigarette smoke extract-induced downregulation of p300 is responsible for the impaired inflammatory cytokine response of macrophages. Cell Signal. 2021;85:110050. 34044126 10.1016/j.cellsig.2021.110050

[29] Le Y, Wang Y, Zhou L, Xiong J, Tian J, Yang X, et al. Cigarette smoke-induced HMGB1 translocation and release contribute to migration and NF-κB activation through inducing autophagy in lung macrophages. J Cell Mol Med. 2020;24:1319–31. 31769590 10.1111/jcmm.14789 PMC6991703

[30] He S, Chen D, Hu M, Zhang L, Liu C, Traini D, et al. Bronchial epithelial cell extracellular vesicles ameliorate epithelial-mesenchymal transition in COPD pathogenesis by alleviating M2 macrophage polarization. Nanomedicine. 2019;18:259–71. 30981817 10.1016/j.nano.2019.03.010

[31] Jiang X, Stockwell BR, Conrad M. Ferroptosis: mechanisms, biology and role in disease. Nat Rev Mol Cell Biol. 2021;22:266–82. 33495651 10.1038/s41580-020-00324-8 PMC8142022

[32] Yu Y, Hai Y, Zhou H, Bao W, Hu X, Gao Y, et al. METTL3 Inhibition suppresses cell growth and survival in colorectal cancer via ASNS downregulation. J Cancer. 2024;15:4853–65. 39132158 10.7150/jca.96760 PMC11310885

[33] Ferrera MC, Labaki WW, Han MK. Advances in chronic obstructive pulmonary disease. Annu Rev Med. 2021;72:119–34. 33502902 10.1146/annurev-med-080919-112707 PMC8011854

[34] Patel N. An update on COPD prevention, diagnosis, and management: the 2024 GOLD report. Nurse Pract. 2024;49:29–36. 38941078 10.1097/01.NPR.0000000000000180

[35] Ritchie AI, Wedzicha JA, Definition. Causes, Pathogenesis, and consequences of chronic obstructive pulmonary disease exacerbations. Clin Chest Med. 2020;41:421–38. 32800196 10.1016/j.ccm.2020.06.007 PMC7423341

[36] Wang Q, Liu S. The effects and pathogenesis of PM2.5 and its components on chronic obstructive pulmonary disease. Int J Chron Obstruct Pulmon Dis. 2023;18:493–506. 37056681 10.2147/COPD.S402122 PMC10086390

[37] Verleden SE, Hendriks JMH, Snoeckx A, Mai C, Mentens Y, Callebaut W, et al. Small airway disease in Pre-Chronic obstructive pulmonary disease with emphysema: A Cross-Sectional study. Am J Respir Crit Care Med. 2024;209:683–92. 38055196 10.1164/rccm.202301-0132OC

[38] Hurst JR, Siddiqui MK, Singh B, Varghese P, Holmgren U, de Nigris E. A systematic literature review of the humanistic burden of COPD. Int J Chron Obstruct Pulmon Dis. 2021;16:1303–14. 34007170 10.2147/COPD.S296696 PMC8121160

[39] Moll M, Silverman EK. Precision approaches to chronic obstructive pulmonary disease management. Annu Rev Med. 2024;75:247–62. 37827193 10.1146/annurev-med-060622-101239

[40] Jiang X, Liu B, Nie Z, Duan L, Xiong Q, Jin Z, et al. The role of m6A modification in the biological functions and diseases. Signal Transduct Target Ther. 2021;6:74. 33611339 10.1038/s41392-020-00450-x PMC7897327

[41] Zhang Y, Wang L, Yan F, Yang M, Gao H, Zeng Y. Mettl3 mediated m6A methylation involved in Epithelial-Mesenchymal transition by targeting SOCS3/STAT3/SNAI1 in cigarette Smoking-Induced COPD. Int J Chron Obstruct Pulmon Dis. 2023;18:1007–17. 37275442 10.2147/COPD.S398289 PMC10239240

[42] Wang L, Yu Q, Xiao J, Chen Q, Fang M, Zhao H. Cigarette smoke Extract-Treated mouse airway epithelial Cells-Derived Exosomal LncRNA MEG3 promotes M1 macrophage polarization and pyroptosis in chronic obstructive pulmonary disease by upregulating TREM-1 via m(6)A methylation. Immune Netw. 2024;24:e3. 38725674 10.4110/in.2024.24.e3 PMC11076299

[43] Fu Y, Dominissini D, Rechavi G, He C. Gene expression regulation mediated through reversible m⁶A RNA methylation. Nat Rev Genet. 2014;15:293–306. 24662220 10.1038/nrg3724

[44] Wu R, Jiang D, Wang Y, Wang X. N (6)-Methyladenosine (m(6)A) methylation in mRNA with A dynamic and reversible epigenetic modification. Mol Biotechnol. 2016;58:450–9. 27179969 10.1007/s12033-016-9947-9

[45] Wei G. RNA m6A modification, signals for degradation or stabilisation? Biochem Soc Trans. 2024;52:707–17. 38629637 10.1042/BST20230574 PMC11088905

[46] Huang H, Weng H, Sun W, Qin X, Shi H, Wu H, et al. Recognition of RNA N(6)-methyladenosine by IGF2BP proteins enhances mRNA stability and translation. Nat Cell Biol. 2018;20:285–95. 29476152 10.1038/s41556-018-0045-z PMC5826585

[47] Zhou H, Sun Q, Feng M, Gao Z, Jia S, Cao L, et al. Regulatory mechanisms and therapeutic implications of insulin-like growth factor 2 mRNA-binding proteins, the emerging crucial m(6)A regulators of tumors. Theranostics. 2023;13:4247–65. 37554271 10.7150/thno.86528 PMC10405845

[48] Yankova E, Blackaby W, Albertella M, Rak J, De Braekeleer E, Tsagkogeorga G, et al. Small-molecule Inhibition of METTL3 as a strategy against myeloid leukaemia. Nature. 2021;593:597–601. 33902106 10.1038/s41586-021-03536-w PMC7613134

[49] Xia H, Wu Y, Zhao J, Cheng C, Lin J, Yang Y, et al. N6-Methyladenosine-modified circSAV1 triggers ferroptosis in COPD through recruiting YTHDF1 to facilitate the translation of IREB2. Cell Death Differ. 2023;30:1293–304. 36828914 10.1038/s41418-023-01138-9 PMC10154389

[50] Lu Z, Yang H, Shao Y, Sun W, Jiang Y, Li J. IGF2BP3-NRF2 axis regulates ferroptosis in hepatocellular carcinoma. Biochem Biophys Res Commun. 2022;627:103–10. 36030651 10.1016/j.bbrc.2022.08.040

[51] Deng L, Di Y, Chen C, Xia J, Lei B, Li N, et al. Depletion of the N(6)-Methyladenosine (m6A) reader protein IGF2BP3 induces ferroptosis in glioma by modulating the expression of GPX4. Cell Death Dis. 2024;15:181. 38429265 10.1038/s41419-024-06486-z PMC10907351

[52] Rochette L, Dogon G, Rigal E, Zeller M, Cottin Y, Vergely C. Lipid peroxidation and iron metabolism: two corner stones in the homeostasis control of ferroptosis. Int J Mol Sci. 2022;24:449. 36613888 10.3390/ijms24010449 PMC9820499

[53] Liu J, Kang R, Tang D. Signaling pathways and defense mechanisms of ferroptosis. FEBS J. 2022;289:7038–50. 34092035 10.1111/febs.16059

[54] He F, Ru X, Wen T. NRF2, a transcription factor for stress response and beyond. Int J Mol Sci. 2020;21:4777. 32640524 10.3390/ijms21134777 PMC7369905

[55] Zhang YG, Yan XF, Liu F, Hao WZ, Cai Y, Liu Y, et al. [Astragalus polysaccharides induces ferroptosis in ovarian adenocarcinoma cells through Nrf2/SLC7A11/GPX4 signaling pathway]. Zhongguo Zhong Yao Za Zhi. 2024;49:6459–67. 39805792 10.19540/j.cnki.cjcmm.20240919.501

[56] Wang X, Wang Y, Huang D, Shi S, Pei C, Wu Y, et al. Astragaloside IV regulates the ferroptosis signaling pathway via the Nrf2/SLC7A11/GPX4 axis to inhibit PM2.5-mediated lung injury in mice. Int Immunopharmacol. 2022;112:109186. 36115280 10.1016/j.intimp.2022.109186

[57] Yu X, He Z, Wang Z, Ke S, Wang H, Wang Q, et al. Brusatol hinders the progression of bladder cancer by Chac1/Nrf2/SLC7A11 pathway. Exp Cell Res. 2024;438:114053. 38663476 10.1016/j.yexcr.2024.114053

