# Supplementary figures and images for: METTL3 mediates m6A methylation of LCN2 through IGF2BP3 to promote ferroptosis in chronic obstructive pulmonary disease
Source: Hereditas. 2025 Dec 26;163:14. doi: 10.1186/s41065-025-00628-9 (PMC12849124; doi:10.1186/s41065-025-00628-9)

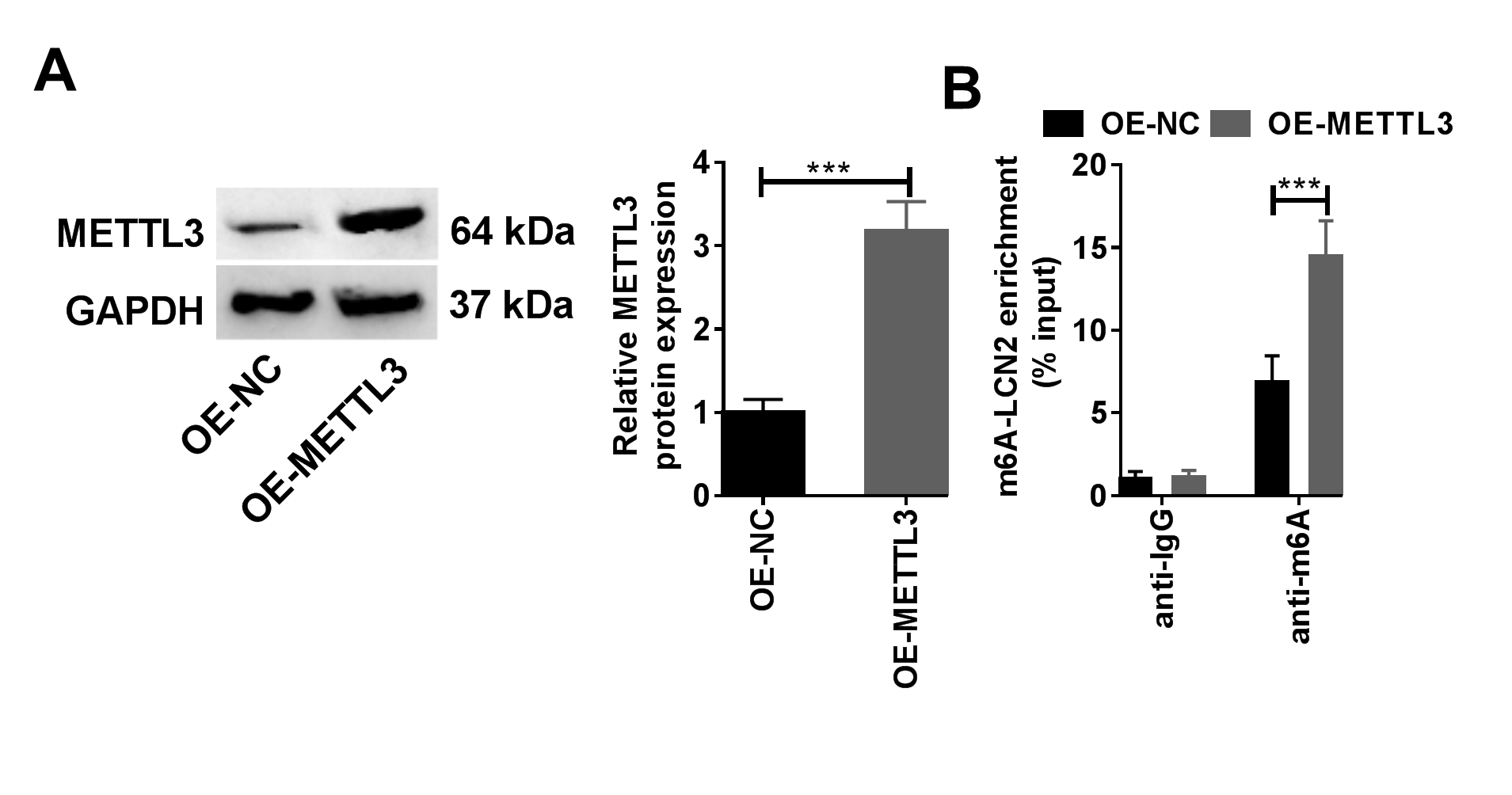

Supplement: Supplementary file 1 — Supplementary Material 1. [file 41065_2025_628_MOESM1_ESM.tif]

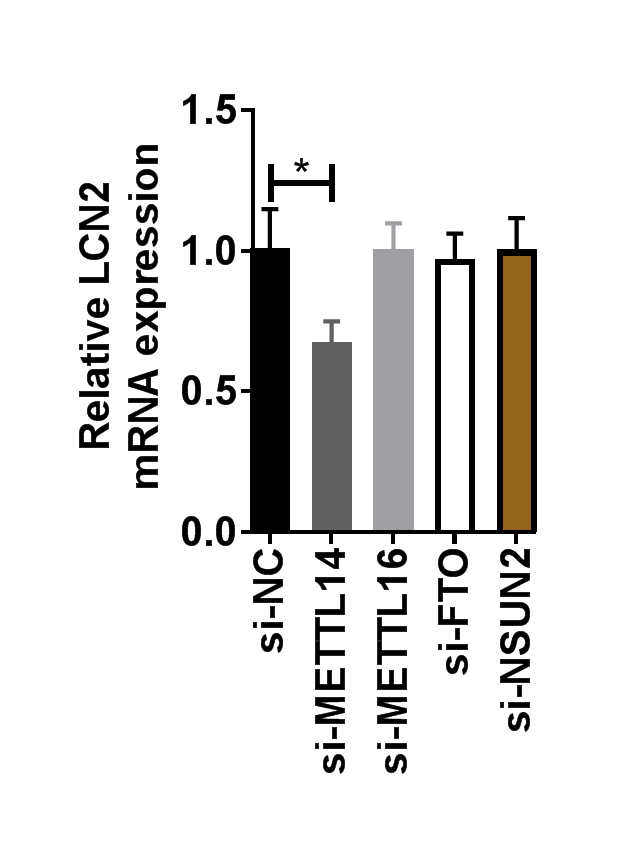

Supplement: Supplementary file 2 — Supplementary Material 2. [file 41065_2025_628_MOESM2_ESM.tif]

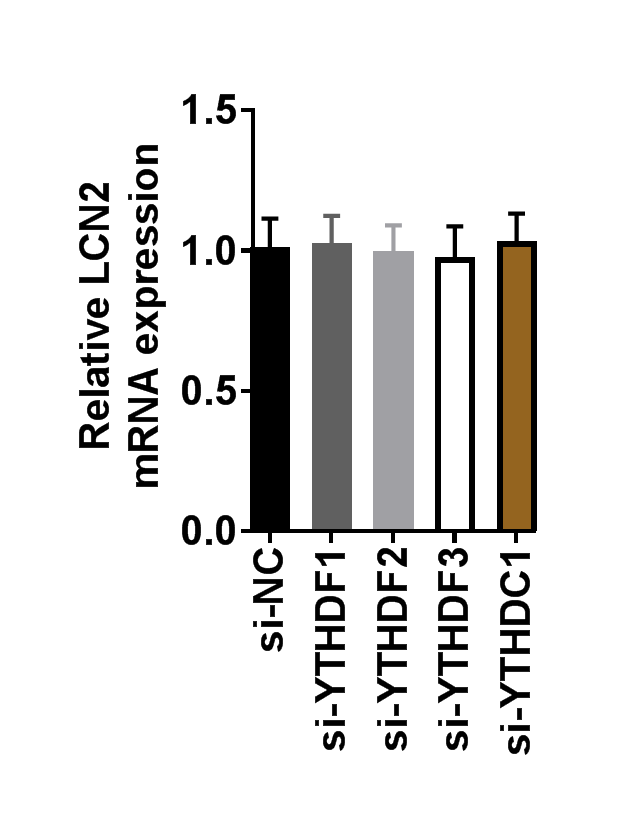

Supplement: Supplementary file 3 — Supplementary Material 3. [file 41065_2025_628_MOESM3_ESM.tif]

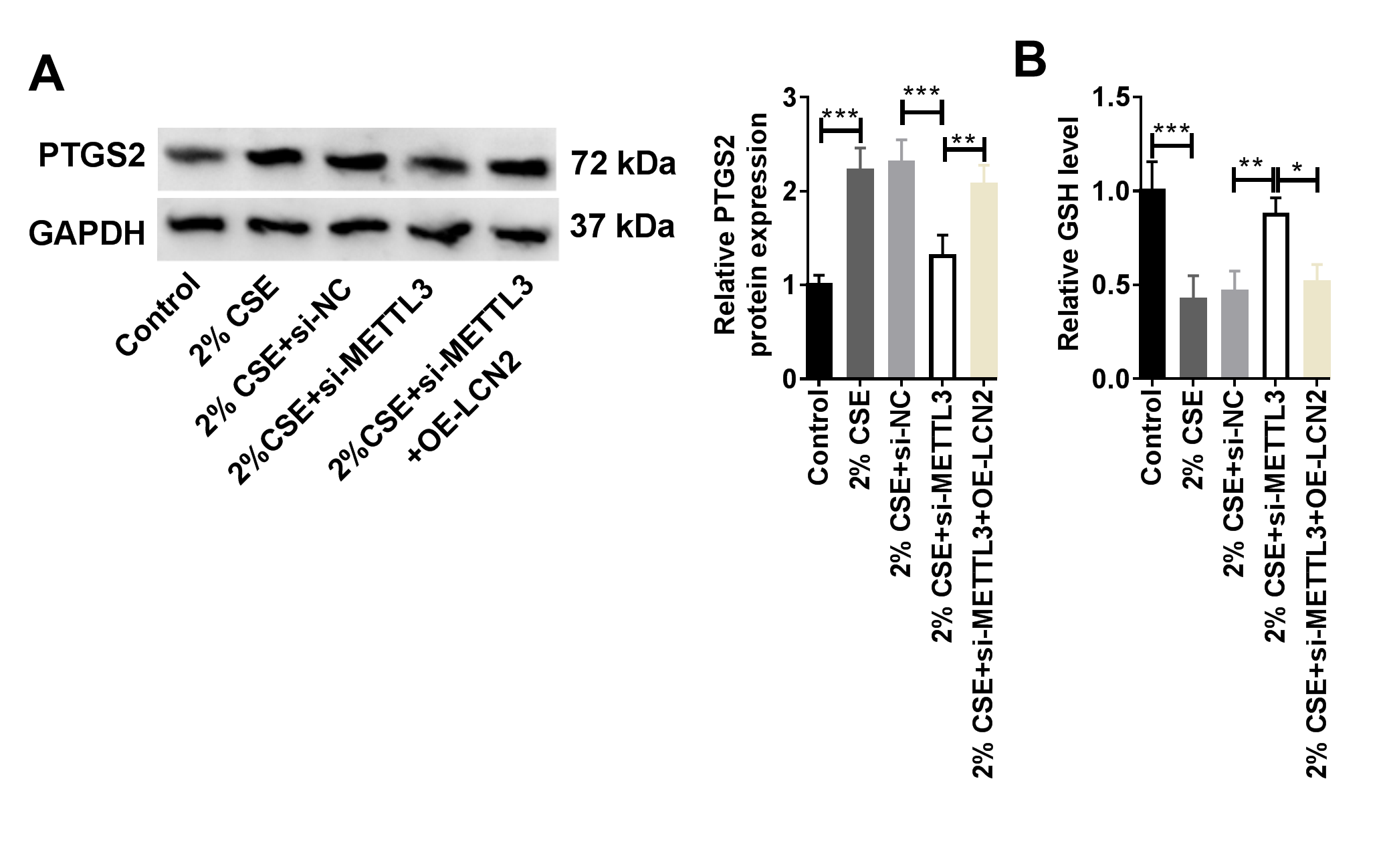

Supplement: Supplementary file 4 — Supplementary Material 4. [file 41065_2025_628_MOESM4_ESM.tif]

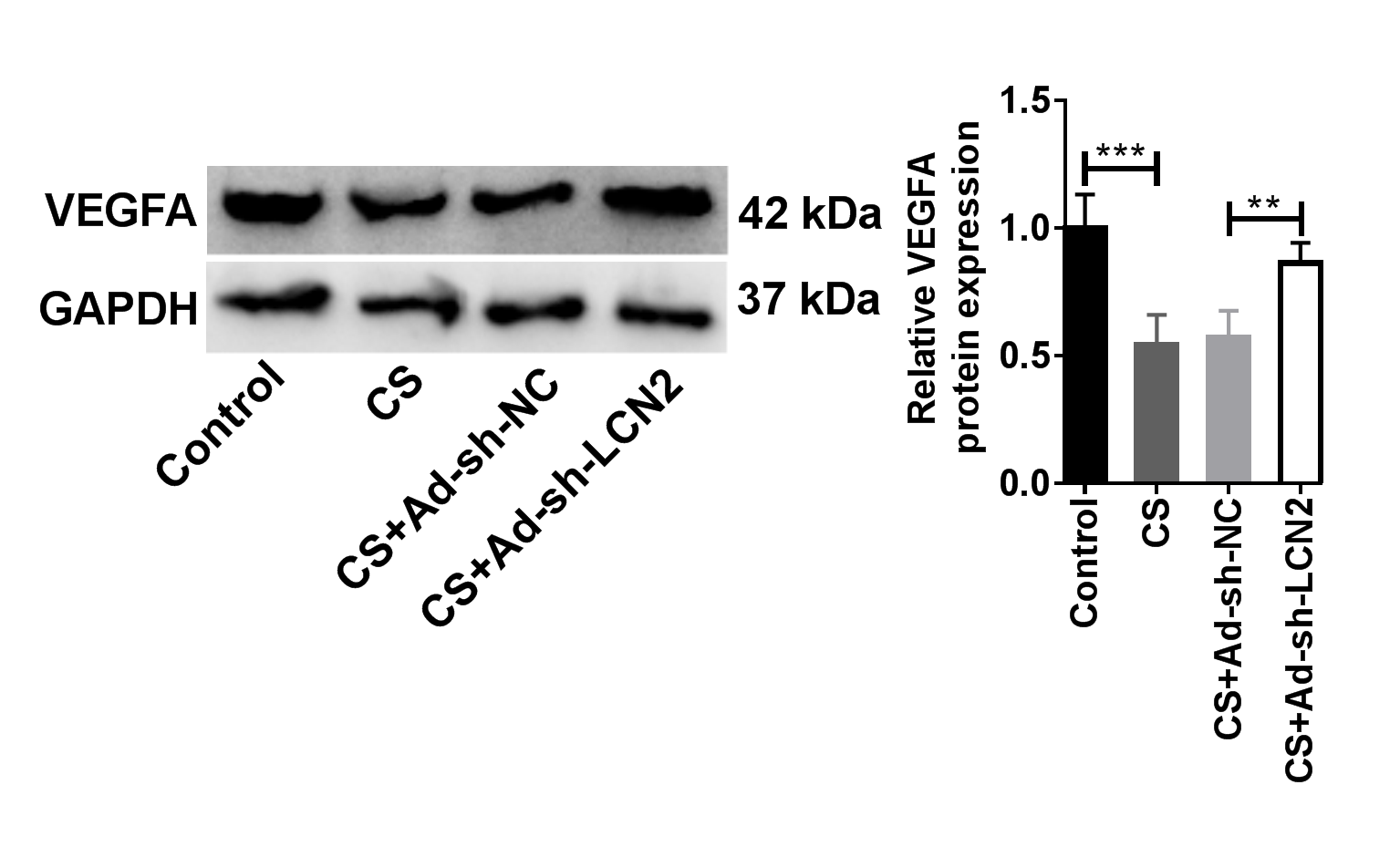

Supplement: Supplementary file 5 — Supplementary Material 5. [file 41065_2025_628_MOESM5_ESM.tif]
