# Supplementary material for: METTL3 mediates m6A methylation of LCN2 through IGF2BP3 to promote ferroptosis in chronic obstructive pulmonary disease
Source: Hereditas. 2025 Dec 26;163:14. doi: 10.1186/s41065-025-00628-9 (PMC12849124; doi:10.1186/s41065-025-00628-9)
Supplement: Supplementary file 6 — Supplementary Material 6. [file 41065_2025_628_MOESM6_ESM.pdf]

The original western blots of Fig1

A

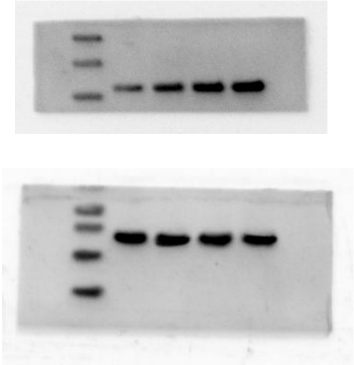

B

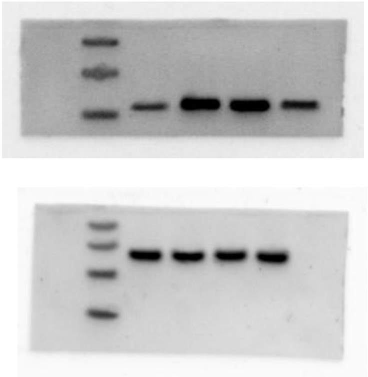

The original western blots of Fig2

D

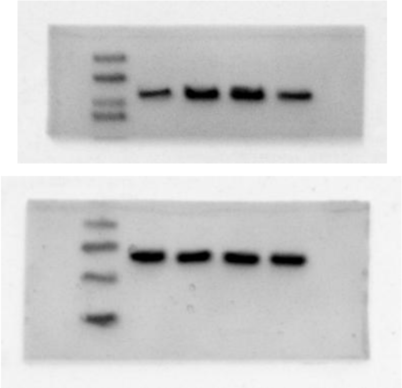

64 kDa METTL3

37 kDa GAPDH

H

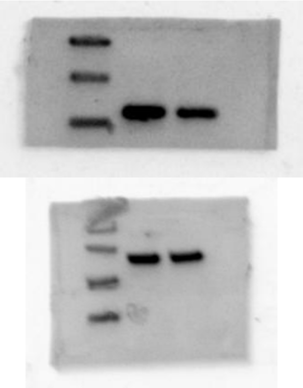

22 kDa LCN2

37 kDa GAPDH

The original western blots of Fig3

A

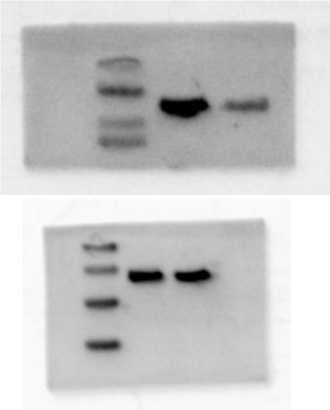

64 kDa IGF2BP1

37 kDa GAPDH

B

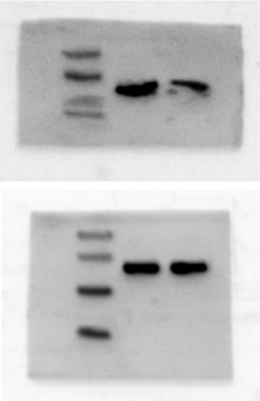

64 kDa IGF2BP2

37 kDa GAPDH

The original western blots of Fig3

C

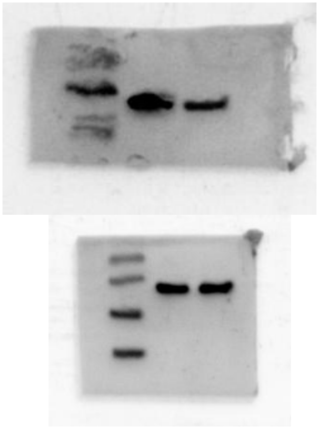

69 kDa IGF2BP3

37 kDa GAPDH

E

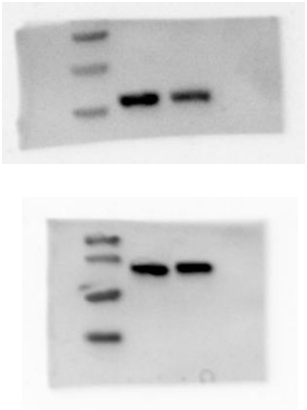

22 kDa LCN2

37 kDa GAPDH

The original western blots of Fig5

A

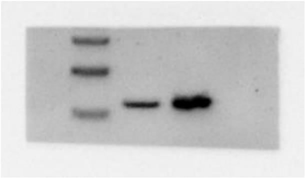

22 kDa LCN2

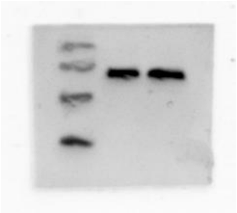

37 kDa GAPDH

The original western blots of Fig6

A

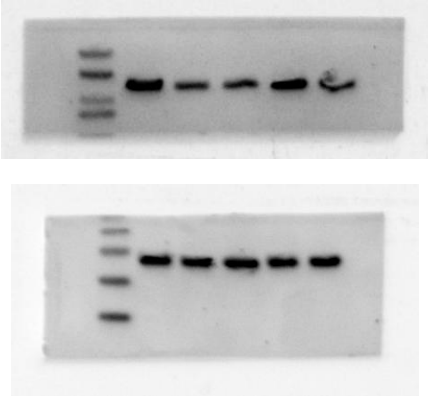

68 kDa NRF2

37 kDa GAPDH

B

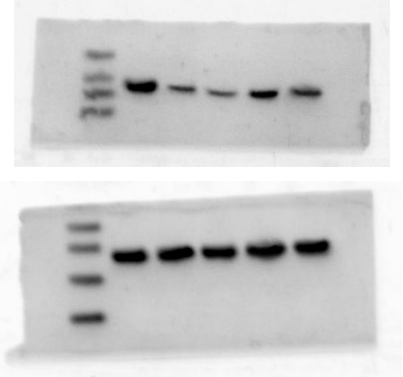

55 kDa SLC7A11

37 kDa GAPDH

C

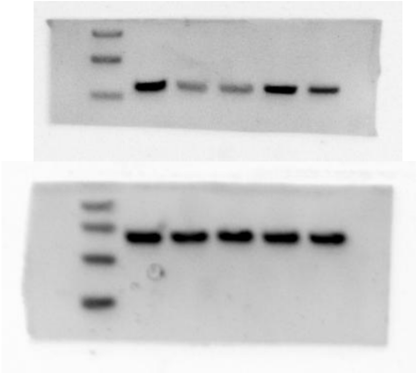

22 kDa GPX4

37 kDa GAPDH

The original western blots of Fig7

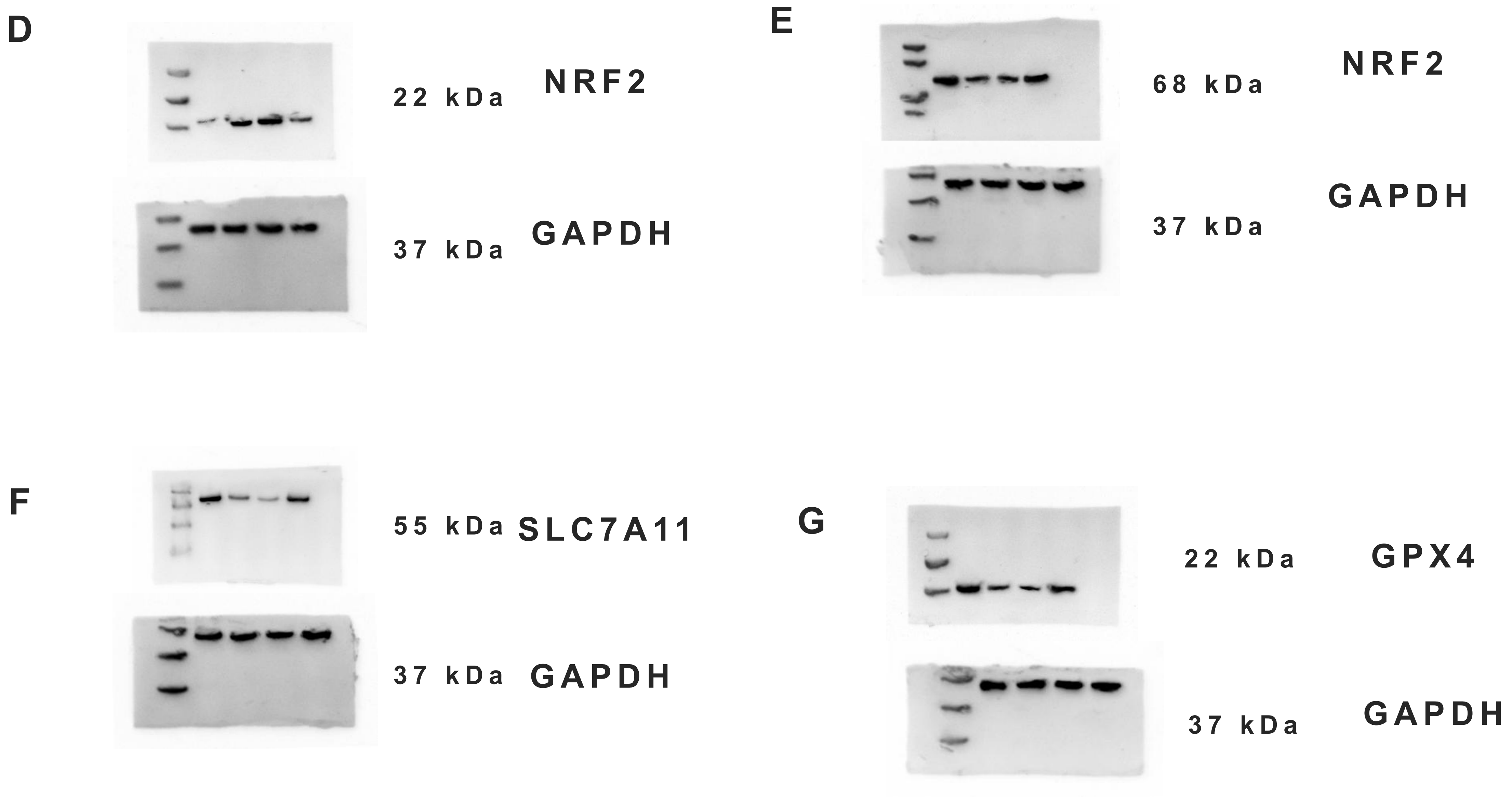

The original western blots of supplementary Figure 1

A

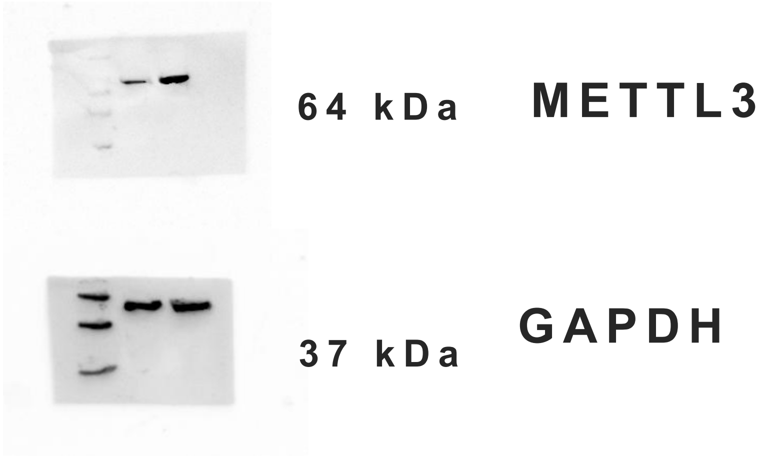

64 kDa METTL3

37 kDa GAPDH

The original western blots of supplementary Figure 4

A

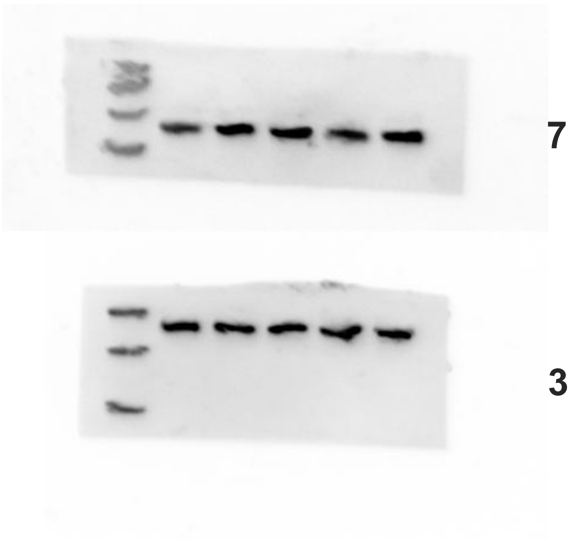

The original western blots of supplementary Figure 5

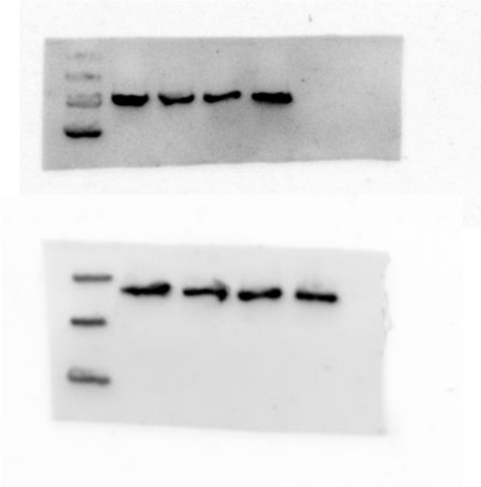

42 kDa VEGFA

37 kDa GAPDH
